# Subacromial decompression versus diagnostic arthroscopy for shoulder impingement: a 5-year follow-up of a randomised, placebo surgery controlled clinical trial

**DOI:** 10.1136/bjsports-2020-102216

**Published:** 2020-10-05

**Authors:** Mika Paavola, Kari Kanto, Jonas Ranstam, Antti Malmivaara, Jari Inkinen, Juha Kalske, Vesa Savolainen, Ilkka Sinisaari, Simo Taimela, Teppo L Järvinen, For the Finnish Shoulder Impingement Arthroscopy Controlled Trial (FIMPACT) Investigators, Jarkko Pajarinen

**Affiliations:** 1 Department of Orthopaedics and Traumatology, Helsinki University Hospital, Helsinki, Finland; 2 TAYS Hatanpää/Department of Orthopedics and Traumatology, Tampere University Hospital, Tampere, Finland; 3 Finnish Centre for Evidence-Based Orthopaedics (FICEBO), Department of Orthopaedics and Traumatology, University of Helsinki, Helsinki, Finland; 4 Mdas AB, Ystad, Sweden; 5 Centre for Health and Social Economics – CHESS, National Institute for Health and Welfare, Helsinki, Finland; 6 Fysios Finlayson, Tampere, Finland; 7 Department of Orthopaedics and Traumatology, Pohjola Hospital, Helsinki, Finland; 8 Terveystalo Healthcare Services, Helsinki, Uusimaa, Finland

**Keywords:** shoulder, acromioplasty, impingement, physiotherapy, placebo, sham, randomised

## Abstract

**Objectives:**

To assess the long-term efficacy of arthroscopic subacromial decompression (ASD) by comparing it with diagnostic arthroscopy (primary comparison), a placebo surgical intervention, and with a non-operative alternative, exercise therapy (secondary comparison).

**Methods:**

We conducted a multicentre, three group, randomised, controlled superiority trial. We included 210 patients aged 35–65 years, who had symptoms consistent with shoulder impingement syndrome for more than 3 months. 175 participants (83%) completed the 5 years follow-up. Patient enrolment began on 1 February 2005 and the 5-year follow-up was completed by 10 October 2018. The two primary outcomes were shoulder pain at rest and on arm activity measured with Visual Analogue Scale (VAS). Minimally important difference (MID) was set at 15. We used a mixed-model repeated measurements analysis of variance with participant as a random factor, the baseline value as a covariate and assuming a covariance structure with compound symmetry.

**Results:**

In the primary intention to treat analysis (ASD vs diagnostic arthroscopy), there were no between-group differences that exceeded the MID for the primary outcomes at 5 years: the mean difference between groups (ASD minus diagnostic arthroscopy) in pain VAS were −2.0 (95% CI −8.5 to 4.6; p=0.56) at rest and −8.0 (−17.3 to 1.3; p=0.093) on arm activity. There were no between-group differences in the secondary outcomes or adverse events that exceeded the MID. In our secondary comparison (ASD vs exercise therapy), the mean differences between groups (ASD minus exercise therapy) in pain VAS were 1.0 (−5.6 to 7.6; p=0.77) at rest and −3.9 (−12.8 to 5.1; p=0.40) on arm activity. There were no significant between-group differences for the secondary outcomes or adverse events.

**Conclusions:**

ASD provided no benefit over diagnostic arthroscopy (or exercise therapy) at 5 years for patients with shoulder impingement syndrome.

## Introduction

Up to 70% of patients suffering from shoulder pain without a preceding traumatic event receive a diagnosis of shoulder impingement or subacromial pain syndrome.^1^ The classic diagnostic sign, subacromial pain while lifting the arm, has been attributed to impingement of the rotator cuff tendons between the humeral head and the undersurface of the acromion. Premised on this rationale, arthroscopic subacromial decompression (ASD), the most commonly performed shoulder surgery,^2^ is believed to relieve symptoms through removal of a bony acromial spur and the resulting decompression of the tendon passage.

A recent BMJ Rapid Recommendation, informed by two linked systematic reviews and/or meta-analyses^3 4^ and a Cochrane systematic review,^5^ concluded with high certainty that in people with painful shoulder impingement, subacromial decompression surgery does not improve pain, function or health-related quality of life compared with placebo surgery or other options that included various forms of physiotherapy, in the short term.^6^ Based on this evidence, the clinical practice guideline panel made a strong recommendation against surgery. However, both the BMJ RapidRec guideline and the Cochrane review panels rated the available evidence on the medium-term to long-term efficacy of subacromial decompression surgery as low quality due to imprecise estimates. The existing long-term evidence consists of 5–10 years follow-up studies comparing ASD to exercise therapy.^7–10^ In three of these unblinded trials, there was no clinically meaningful benefit,^7 8 10^ while one trial suggested a benefit of ASD.^9^ Updates to the BMJ RapidRec guideline and the Cochrane review are pending low risk of bias evidence on the long-term outcomes on subacromial decompression surgery. According to the Cochrane review,^5^ the fate of this enormously popular surgical procedure now hangs on the thread of this study as it is the only ongoing low risk of bias trial assessing the long-term efficacy of subacromial decompression surgery.

We conducted a multicentre, randomised, double blind, placebo surgery controlled trial to assess the long-term (5 years) efficacy of ASD in patients with symptoms consistent with shoulder impingement syndrome. Our trial also included an exercise therapy as a non-operative alternative, to allow comparison between ASD and exercise therapy in a more pragmatic setting.

## Methods

### Trial design

We conducted this superiority trial at three orthopaedic clinics in Finland from 1 February 2005 to 10 October 2018. Details of the trial design and conduct, and results at 2-year follow-up have been published.^11 12^ The participants in the two surgical groups and the people who collected the data were unaware of the study group assignments.

The trial was conducted in accordance with the Declaration of Helsinki. All patients gave written informed consent. On entering the study, they were unequivocally informed that they might undergo diagnostic arthroscopy and that they would be allowed to consider crossing over to ASD if they did not have adequate relief of symptoms, preferably no sooner than 6 months after randomisation.

### Participants

We enrolled patients aged 35–65 years who had subacromial pain for more than 3 months that was unresponsive to conventional conservative treatment, and clinical findings consistent with shoulder impingement syndrome. All patients had MRI with intra-articular contrast to exclude a rotator cuff tear. Detailed eligibility criteria are provided in the box 1.

Box 1 **Inclusion and exclusion criteria**

**Inclusion criteria**

1. Adult men or women ages 35–65 years.
2. Subacromial pain for greater than 3 months with no relief from non-operative means (physiotherapy, non-steroidal anti-inflammatory medication, corticosteroid injections and rest).
3. Pain provoked by abduction and positive painful arc sign.
4. Positive impingement test (temporary relief of pain by subacromial injection of lidocaine).
5. Pain in at least two out of three of isometric tests (abduction 0° and 30° or external rotation).
6. Provision of informed consent from the participant.
7. Ability to speak, understand and read in the language of the clinical site.

**Exclusion criteria**

1. Full thickness tear of the rotator cuff tendons diagnosed on clinical examination (marked weakness in any of the examined muscles) or MRI with intra-articular contrast (MR arthrography).
2. Osteoarthritis of the glenohumeral and/or acromioclavicular joint diagnosed on clinical examination or on X-rays.
3. Substantial calcific deposits in the rotator cuff tendons found in the preoperative imaging.
4. Previous surgical procedure on the affected shoulder.
5. Evidence of shoulder instability (positive apprehension/positive sulcus sign).
6. Symptomatic cervical spine pathology.
7. History of alcoholism, drug abuse, psychological or psychiatric problems that are likely to invalidate informed consent.
8. Patient declined to participate.

### Randomisation and blinding

In an attempt to obtain three balanced study groups of similar group size, we planned a twofold, sequential randomisation: patients were first randomised to surgery or exercise therapy in 2:1 ratio during the baseline appointment. Patients randomised to exercise therapy started standardised physiotherapy within 2 weeks of the baseline appointment, whereas those allocated to surgery were scheduled for surgery with the aim of carrying out the procedure within 12 weeks of this first randomisation. Patients allocated to surgery first had a diagnostic arthroscopy to rule out a rotator cuff tendon tear and other pathology requiring surgical treatment. If a patient had a full-thickness or a partial-thickness rotator cuff tear large enough to require repair (grade III),^13^ the patient was excluded, and the rotator cuff tear was repaired. Patients with a partial tear not requiring repair (grade I and II) were included. If the eligibility of the patient was confirmed at the diagnostic arthroscopy, the second randomisation was carried out by opening an envelope containing the either ASD or diagnostic arthroscopy in 1:1 ratio. Only the orthopaedic surgeon and other staff in the operating room were aware of the surgical group assignment, and they did not participate in further treatment or follow-up of the patient.

Randomisation was carried out using sequentially numbered sealed opaque envelopes. Separate randomisation lists for each centre, with blocks varying randomly in size, were prepared by a statistician with no involvement in the clinical care of participants in the trial.

### Study intervention

#### Exercise therapy

A physiotherapist supervised, progressive, individually designed exercise therapy was started within 2 weeks of randomisation. Participants completed 15 visits to physiotherapists and a standardised home exercise protocol (the detailed exercise therapy protocol is available in online supplementary appendix). All study physiotherapists supervising the exercise therapy were specially trained to conduct our protocol.

10.1136/bjsports-2020-102216.supp1 Supplementary data

#### Diagnostic arthroscopy

**Figure 1:**
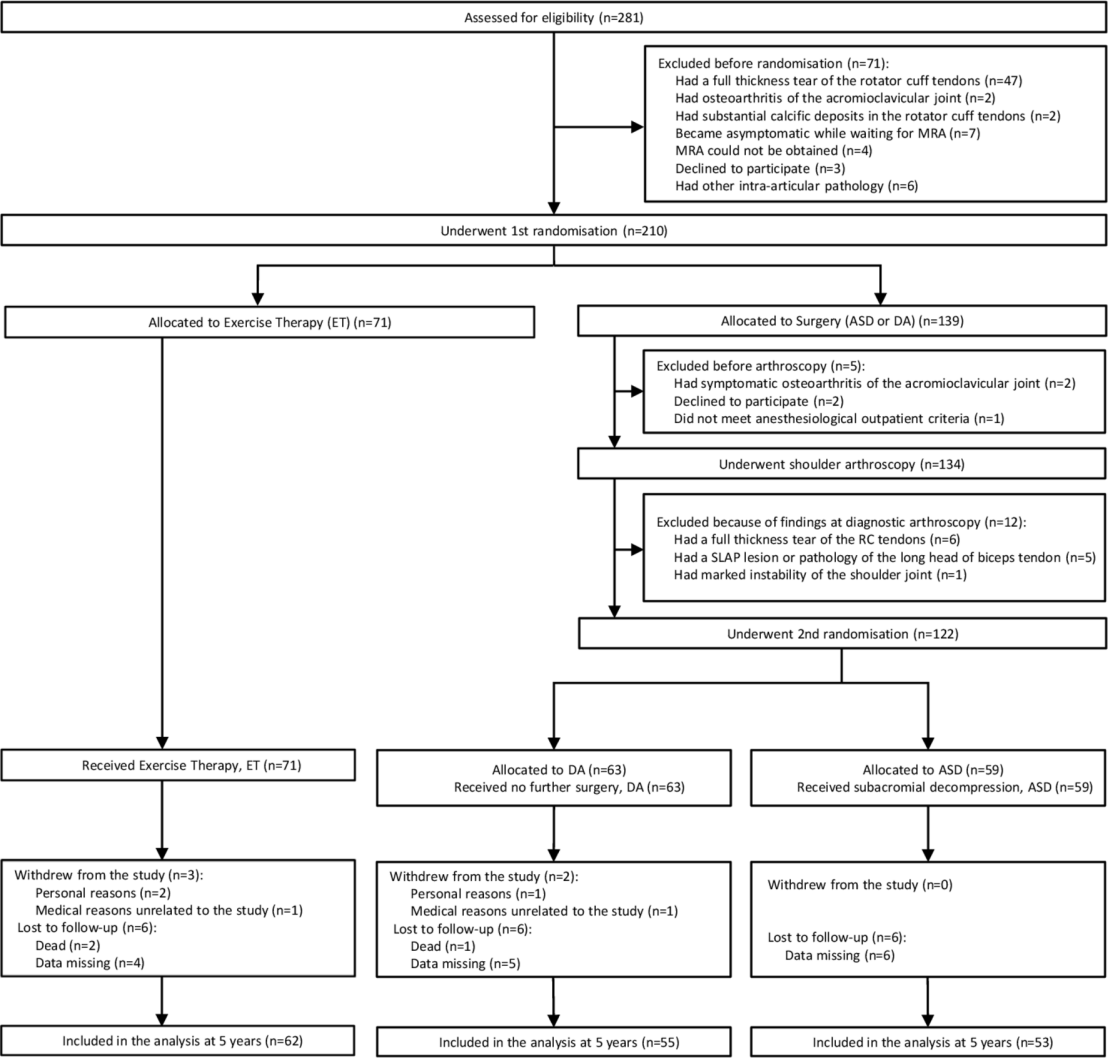
Study flow chart. Full details of unblinding, treatment conversions and reoperations are provided in online supplementary appendix table S5. ASD, arthroscopic subacromial decompression; MRA, MR arthrography; RC, rotator cuff; SLAP, superior labrum anterior–posterior.

We carried out arthroscopic examination of the glenohumeral joint and subacromial space with the use of standard posterior and lateral portals and a 4 mm arthroscope with the participant under general anaesthesia, usually supplemented with an interscalene brachial plexus block. We performed an intra-articular and subacromial assessment of the rotator cuff integrity. If the rotator cuff insertion could not be otherwise visualised, the subacromial bursal tissue was bluntly stretched with a trochar or minimally resected. If arthroscopic examination revealed any pathology requiring intervention other than ASD, the patient was excluded from the trial (figure 1). Once eligibility was confirmed, participants were randomly assigned to either ASD or diagnostic arthroscopy. For those allocated to the diagnostic arthroscopy group, the operation was terminated. To ensure concealment of the allocation from participants and the staff other than those in the operating theatre, the diagnostic arthroscopy participants remained in the operating theatre for the time required to perform subacromial decompression.

#### Arthroscopic subacromial decompression

For patients allocated to ASD after diagnostic arthroscopy, we continued the surgery by performing a standard ASD procedure—a subacromial bursectomy and resection of bony spurs and the projecting anterolateral undersurface of the acromion with a shaver, burr and/or electrocoagulation.^14^

### Postoperative rehabilitation

In the surgically treated patients (ASD and diagnostic arthroscopy groups), postoperative rehabilitation was identical. It consisted one outpatient visit to a study physiotherapist, blind to the group assignment, for guidance and instructions for home exercises.

### Outcome measures

Given that the pathognomonic clinical sign of shoulder impingement syndrome is subacromial shoulder pain, especially at night and while lifting the arm, our two primary outcome measures were shoulder pain at rest and shoulder pain on arm activity at 5 years. We used a Visual Analogue Scale (VAS) ranging from 0 (no pain) to 100 (extreme pain) to measure the outcomes. We considered 15 points as the minimal important difference (MID).^15^

The secondary outcomes were two shoulder function assessment instruments, the Constant-Murley score (CM) and the simple shoulder test (SST), and two health-related quality of life instruments, the SF-36(R) Health Survey^16^ and the 15D.^17^ MID was set at 17 points as for the CM^18^ and two points for SST.^19^ Patients’ global assessment of satisfaction to the treatment was elicited with this question: ‘Are you satisfied with the treatment you have received?’ We used a VAS scale ranging from 0 (completely disappointed) to 100 (very satisfied). Patients’ satisfaction with the treatment outcome was elicited with a question: ‘How satisfied are you with the outcome of your treatment?’ on a 5-item scale. Patients who reported very satisfied or satisfied were categorised as ‘Responders’.

Questionnaires were administered at baseline and 3, 6, 12, 24 months and 5 years after randomisation. The follow-up questionnaires also included a separate section on adverse events (AEs). AEs were defined as untoward medical occurrences that did not necessarily have a causal relationship with the treatment administered. Serious AEs were events with the potential for substantial disability/incapacity and/or requiring inpatient hospital care or prolonged hospital care, or were life threatening, or resulted in death. In this paper, we report AEs that we deemed to be directly related to the treatments given.

To assess whether patients in the placebo surgery group were more likely than patients in the ASD group to guess that they had undergone a placebo procedure, all surgically treated participants were asked at the 3 months follow-up which procedure (ASD or diagnostic arthroscopy) they thought they had had.

### Statistical analysis and sample size calculation

We powered the study to detect a difference of at least the MID (15 points^15^) in the two primary outcomes between the ASD and diagnostic arthroscopy groups. For the study to have 90% power to show a minimal clinically important advantage of ASD over diagnostic arthroscopy, under the assumption of a two-sided type 1 error rate of 5%, we planned to recruit 70 participants per group.

The trial was primarily designed to ascertain whether ASD was superior to diagnostic arthroscopy for pain reduction (two primary outcomes) at 5 years after the procedure (the primary confirmatory comparison). We also included a pragmatic comparison of the relative benefits of ASD vs exercise therapy (the secondary exploratory comparison), at 5 years after the procedure, with the two primary outcomes. All analyses were performed according to the previously published statistical analysis plan^12^ by an independent statistician.

We quantified the treatment effect on an intention to treat (ASD vs diagnostic arthroscopy comparison) or full analysis set (ASD vs exercise therapy comparison) basis as the difference between the groups in pain scores (VAS), CM score, simple shoulder test score, 15D score and SF-36 score with the associated 95% CIs and p values at 5 years after the primary randomisation. In intention-to-treat and full analysis set analyses, the participants were included as randomised. We used a mixed-model repeated measurements analysis of variance with participant as a random factor (repeated measurements at 3, 6, 12, 24 months and 5 years), the baseline value as a covariate, and assuming a covariance structure with compound symmetry. As the mixed-model repeated measurements analysis of variance allows for analysis of unbalanced data sets without imputation, we analysed all available data, the full analysis set. The missingness of the outcome data at different time points is shown in the online supplementary appendix table S8. We fitted the mixed-model repeated measurements model by using the mixed procedure in Stata and used Satterthwaite’s method to calculate the df. We used generalised estimating equation logistic regression analysis to analyse categorical variables. We compared the frequencies of patients who reported satisfaction or subjective improvement, the proportions of responders and non-responders based on patients’ satisfaction with the treatment outcome, and the incidence of treatment group unblindings, treatment conversions, and reoperations between the two groups at 5 years.

To safeguard against potential multiplicity effects^20^ in the primary comparison, we required a statistically significant treatment effect for both primary outcome variables. All secondary analyses were supportive, exploratory and/or hypothesis-generating. We carried out two sensitivity analyses (the per protocol and as treated) with the same principles as the intention-to-treat and full analysis set analyses. The per-protocol population is the subset of the intention to treat population who received the treatment they were randomised to and who did not receive any other treatment, that is, the patients with a treatment conversion have been excluded (ASD: n=59, diagnostic arthroscopy: n=54). The as treated population is defined according to the treatment the participants received, that is, the nine participants who originally received diagnostic arthroscopy and the 14 participants who originally received exercise therapy, but due to persistent symptoms requested unblinding and subsequently received ASD, have been included in the ASD population (ASD: n=83, diagnostic arthroscopy: n=54). We considered a p value of 0.05 to indicate statistical significance. Stata V.15.1 (StataCorp, USA) was used for all statistical analyses.

### Patient involvement

No patients were involved in designing the study, nor were they involved in developing plans for recruitment, design or implementation of the study. No patients were asked to advise on interpretation or writing up of results. When the results of this randomised controlled trial are published, a lay information flyer with final results will be sent to the recruiting centres for dissemination to the trial participants.

## Results

### Characteristics of the patients

Of the 281 eligible patients, 71 were excluded (figure 1). A total of 210 patients underwent the first randomisation; 71 were assigned to exercise therapy and 139 to surgery. Of those allocated to surgery (n=139), another 17 were excluded before the second randomisation; 59 patients received ASD and 63 received diagnostic arthroscopy. Over the course of the 5 years follow-up, three patients withdrew, four could not be reached and two died in the exercise therapy group; five could not be reached and one died in the diagnostic arthroscopy group; and six could not be reached in the ASD group. This left us with 62, 55 and 53 participants to be included in the analysis in the exercise therapy, diagnostic arthroscopy and ASD groups at 5 years, respectively (figure 1). The study groups were well balanced on all baseline characteristics (table 1). The patients who withdrew from the study (n=5) were similar to those who underwent randomisation with respect to primary outcome measures at baseline.

**Table 1:** Baseline characteristics of the participants according to study group

Characteristics	Arthroscopic subacromial decompression (n=59)	Diagnostic arthroscopy (n=63)	Exercise therapy (n=71)
Age, years	50.5 (7.3)	50.8 (7.6)	50.4 (6.6)
Female, n (%)	42 (71)	46 (73)	47 (66)
Dominant hand affected, n (%)	35 (59)	36 (57)	46 (65)
Duration of symptoms, months	18 (14)	18 (19)	22 (23)
Able to work normally regardless of shoulder symptoms, n (%)	27 (46)	31 (49)	35 (49)
Visual analogue scale score, at rest*	41.3 (25.8)	41.6 (25.5)	41.7 (27.5)
Visual Analogue Scale (VAS) score, on arm activity*	71.2 (23.6)	72.3 (21.7)	72.4 (20.8)
Constant-Murley score†	32.2 (15.8)	31.7 (14.0)	35.2 (16.2)
Simple shoulder test score‡	4.9 (2.9)	4.9 (2.9)	4.8 (2.7)
15D score§	0.89 (0.06)	0.89 (0.07)	0.88 (0.08)
SF-36 score¶			
Physical health	74.3 (12.5)	74.1 (13.1)	75.7 (10.1)
Mental health	79.4 (14.2)	77.9 (16.7)	75.6 (18.2)

Data are presented as mean (SD) unless otherwise indicated.

*Shoulder pain at rest and at activity was assessed on a 100 mm VAS of 0–100, with 0 denoting no pain and 100 denoting extreme pain.

†Scoring system for evaluation of various shoulder disorders consisting of both objective (range of motion and strength) and subjective measurements (pain assessment, work load and leisure time activities), summarised in a score between 0 and 100; higher score indicates better shoulder function.

‡Based on 12 questions with yes (1) or no (0) response options; maximum score is 12, indicating normal shoulder function; minimum score of 0 points indicates severely diminished shoulder function.

§Generic health-related quality of life instrument comprising 15 dimensions; maximum score is 1 (full health), and minimum score is 0 (death).

¶Generic health-related quality of life instrument to quantify the physical, functional and psychological aspects of health-related quality of life. It consists of 36 questions in eight subscales that assess physical, functional, social and psychological well-being. Score ranges from 0 to 100, where a higher score is associated with better health.

### Primary comparison: ASD versus diagnostic arthroscopy

#### Primary outcomes

**Figure 2:**
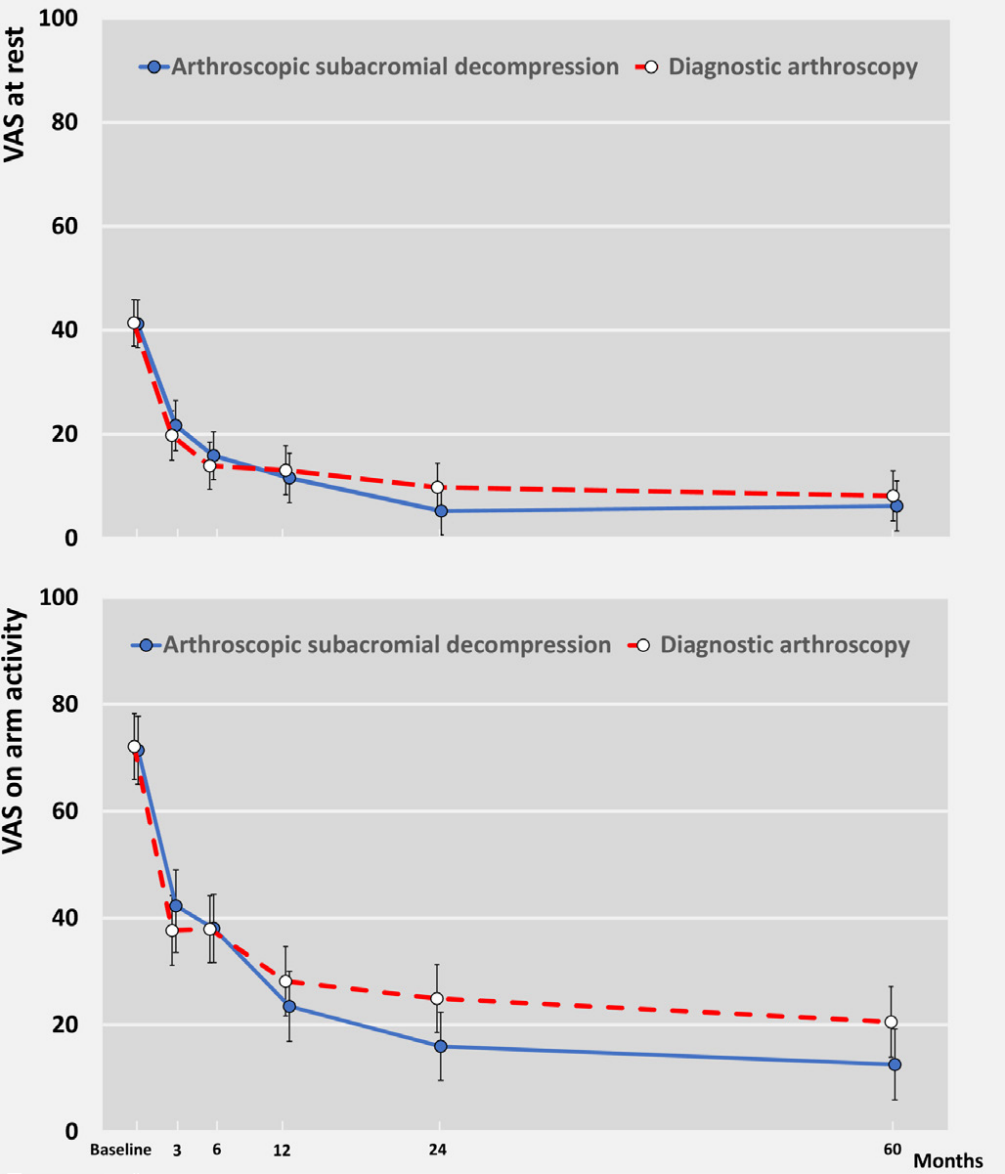
Primary outcomes of primary comparison at baseline and at 3, 6, 12, 24 months and 5 years follow-up. Visual Analogue Scale (VAS) shoulder pain scores at rest and on arm activity over 5 years follow-up period are shown. VAS range from 0 to 100, with higher values indicating more severe pain. data are mean (95% CI) shown at follow-up time points

There was marked improvement from baseline to 5 years for both primary outcomes in the ASD and diagnostic arthroscopy groups (mean change for ASD 35.0 at rest and 58.8 on activity; for diagnostic arthroscopy 33.4 at rest and 51.9 on activity) (figure 2 and table 2). There were no significant between group differences in VAS pain at rest (mean difference, ASD minus diagnostic arthroscopy, −2.0, 95% CI −8.5 to 4.6; p=0.56) or VAS pain on arm activity (−8.0 to –17.3 to 1.3; p=0.093) (figure 2, table 2 and online supplementary appendix table S1). The results remained unaltered in the prespecified sensitivity (as treated and per-protocol) analyses (online supplementary appendix tables S1 and S3).

**Table 2:** Primary comparison arthroscopic subacromial decompression (ASD) versus diagnostic arthroscopy (DA): outcomes of the trial at 5 years follow-up

	ASD (n=53)	DA (n=55)	Between group difference, ASD versus DA	P value
Primary outcomes				
Visual Analogue Scale (VAS) score, at rest	6.2 (1.6 to 10.8)	8.2 (3.5 to 12.8)	−2.0 (−8.5 to 4.6)	0.56
VAS score, on arm activity	12.4 (5.8 to 19.0)	20.4 (13.8 to 26.9)	−8.0 (−17.3 to 1.3)	0.093
Secondary outcomes				
Constant-Murley score	82.8 (78.4 to 87.3)	75.7 (71.3 to 80.1)	7.1 (0.9 to 13.4)	0.025
Simple shoulder test score	10.7 (10.1 to 11.3)	10.3 (9.7 to 11.0)	0.3 (−0.5 to 1.2)	0.45
15D score	0.90 (0.89 to 0.92)	0.92 (0.90 to 0.93)	−0.01 (−0.03 to 0.01)	0.32
SF-36 score				
Physical health	84.0 (80.6 to 87.4)	85.4 (82.0 to 88.8)	−1.4 (−6.1 to 3.4)	0.58
Mental health	79.9 (76.9 to 82.9)	80.1 (77.0 to 83.2)	−0.2 (−4.5 to 4.1)	0.92
Proportion of patients able to return to previous leisure activities*	0.91 (0.83 to 0.98)	0.86 (0.76 to 0.95)	0.05 (−0.07 to 0.17)	0.40
Proportion of responders†	0.99 (0.95 to 1.0)	0.91 (0.84 to 0.99)	0.07 (−0.01 to 0.15)	0.080
Patients’ satisfaction with treatment‡	89.7 (84.5 to 94.9)	85.7 (80.6 to 90.8)	4.0 (−3.2 to 11.3)	0.28
No (%) complications and adverse effects§	3 (5)	2 (3)	–	–

Values are means with 95% CIs unless otherwise indicated. A lower score indicates the desired (better) treatment outcome in pain VAS score and complications, while a higher score indicates the same in all other outcomes. Between-group differences may not exactly equal the difference in changes in score between the ASD and diagnostic arthroscopy groups because of the adjustment for baseline imbalance in the mixed-effects model.

*Patients ability to return to previous leisure activities was assessed with the following question: ‘Have you been able to return to your previous leisure activities?’ (‘yes’ or ‘no’).

†Patients’ satisfaction with the treatment outcome was elicited with a question: ‘How satisfied are you with the outcome of your treatment?’ on a 5-item scale. Patients who reported very satisfied or satisfied were categorised as ‘Responders’.

‡Patients’ global assessment of satisfaction to the treatment was elicited with this question: ‘Are you satisfied with the treatment you have received?’ We used a VAS ranging from 0 (completely disappointed) to 100 (very satisfied).

§Complications directly related to the interventions were registered.

#### Secondary and other outcomes

There was a statistically significant between group difference in CM score in favour of ASD (7.1, 0.9 to 13.4; p=0.025) (table 2 and online supplementary appendix table S2). The mean difference did not exceed the threshold of 17 for MID pre-specified in our protocol.^11^ We found no significant between group differences in any of the other secondary outcomes (table 2 and online supplementary appendix table S2). Participants in the diagnostic arthroscopy group were no more likely than participants in the ASD group to guess that they had undergone a placebo procedure (22/53 (42%) vs 21/54 (39%), respectively; p=0.85).

#### Unblinding of treatment allocation, treatment conversions and reoperations

Six out of 59 participants in the ASD group and 11 out of 63 participants in the diagnostic arthroscopy group (p=0.25) reported persistent symptoms after surgery, sufficiently severe to lead to unblinding of the study group assignment. Two participants in the ASD group had a reoperation: one had manipulation under anaesthesia, the other had acromioclavicular resection and later manipulation under anaesthesia. In the diagnostic arthroscopy group, 10 participants had a reoperation (seven ASDs, two ASDs coupled with other procedures, and one repair of a traumatic supraspinatus tendon rupture). Details of unblindings, treatment conversions and reoperations are shown in online supplementary appendix table S4.

#### Complications and AEs

One participant in the diagnostic arthroscopy group had temporary swelling in the brachial area related to a brachial plexus block. Three participants in the ASD group and one participant in the diagnostic arthroscopy group developed a frozen shoulder (table 2). No other complications directly related to the interventions were registered.

### The secondary comparison: ASD versus exercise therapy

#### Primary outcomes

**Figure 3:**
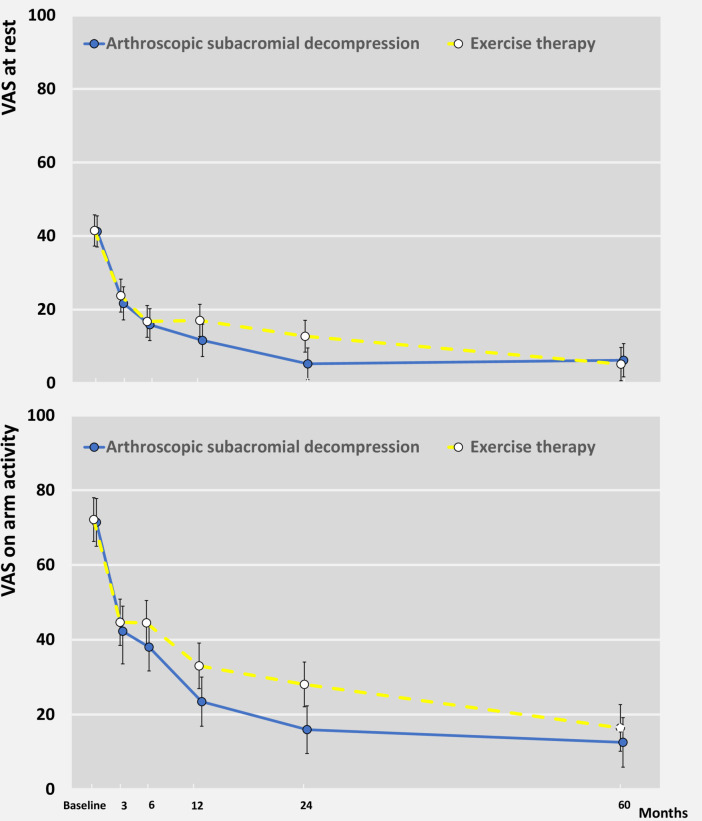
Primary outcomes of secondary comparison at baseline and at 3, 6, 12, 24 months and 5 years follow-up. Visual Analogue Scale (VAS) shoulder pain scores at rest and on arm activity over 5 years follow-up period are shown. VAS range from 0 to 100, with higher values indicating more severe pain. Data are mean (95% CI) shown at follow-up time points.

There was marked improvement from baseline to 5 years for both primary outcomes in the ASD and exercise therapy groups (mean change for ASD at rest 35.0 and on activity 58.6; for exercise therapy at rest 36.4 and on activity 55.9) (figure 3, table 3 and online supplementary appendix table S5). We found no significant between-group differences in VAS pain at rest (1.0,–5.6 to 7.6; p=0.77) or VAS on arm activity (−3.9 to –12.8 to 5.1; p=0.40). The results remained unaltered in the prespecified sensitivity (as treated and per-protocol) analyses (online supplementary appendix table S7).

**Table 3:** Secondary comparison arthroscopic subacromial decompression (ASD) versus exercise therapy (ET): outcomes of the trial at 5 years follow-up

	ASD (n=53)	ET (n=62)	Between-group difference, (ASD versus ET)	P value
Primary outcomes				
Visual Analogue Scale (VAS) score, at rest	6.3 (1.5 to 11.0)	5.3 (0.8 to 9.8)	1.0 (−5.6 to 7.6)	0.77
VAS score, on arm activity	12.6 (6.1 to 19.2)	16.5 (10.3 to 22.6)	−3.9 (−12.8 to 5.1)	0.40
Secondary outcomes				
Constant-Murley score	83.7 (79.2 to 88.1)	79.8 (75.6 to 84.1)	3.9 (−2.3 to 10.0)	0.22
Simple shoulder test score	10.6 (10.0 to 11.3)	10.7 (10.1 to 11.3)	0.0 (−0.9 to 0.8)	0.94
15D score	0.90 (0.89 to 0.91)	0.91 (0.90 to 0.93)	0.01 (−0.03 to 0.01)	0.32
SF-36 score				
Physical health	84.3 (81.2 to 87.3)	87.5 (84.7 to 90.4)	–3.3 (−7.4 to 0.9)	0.13
Mental health	79.2 (75.9 to 82.4)	81.9 (78.8 to 84.9)	−2.7 (−7.2 to 1.8)	0.23
Proportion of patients able to return to previous leisure activities*	0.91 (0.83 to 0.98)	0.80 (0.70 to 0.90)	0.11 (−0.02 to 0.23)	0.067
Proportion of responders†	0.98 (0.95 to 1.0)	0.94 (0.88 to 1.0)	0.04 (−0.03 to 0.11)	0.32
Patients’ satisfaction with treatment‡	89.7 (84.4 to 95.0)	86.8 (81.8 to 91.7)	3.0 (−4.3 to 10.2)	0.42
No (%) complications and adverse effects§	3 (5)	3 (4)	–	–

Values are means with 95% CIs unless otherwise indicated. A lower score indicates the desired (better) treatment outcome in pain VAS score and complications, while a higher score indicates the same in all other outcomes. Between-group differences may not exactly equal the difference in changes in score between the ASD and diagnostic arthroscopy groups because of the adjustment for baseline imbalance in the mixed-effects model.

*Patients ability to return to previous leisure activities was assessed with the following question: ‘Have you been able to return to your previous leisure activities?’ (‘yes’ or ‘no’).

†Patients’ satisfaction with the treatment outcome was elicited with a question: ‘How satisfied are you with the outcome of your treatment?’ on a 5-item scale. Patients who reported very satisfied or satisfied were categorised as ‘Responders’.

‡Patients’ global assessment of satisfaction to the treatment was elicited with this question: ‘Are you satisfied with the treatment you have received?’ We used a VAS ranging from 0 (completely disappointed) to 100 (very satisfied).

§Complications directly related to the interventions were registered.

#### Secondary and other outcomes

We found no significant between group differences in any of the secondary outcomes (table 3 and online supplementary appendix table S6).

#### Unblinding of treatment allocation and crossovers

Sixteen participants out of 71 who were initially assigned to exercise therapy reported persistent symptoms, sufficiently severe to require unblinding. Fifteen participants subsequently had ASD and one participant had acromioclavicular resection. Three reoperations were performed (online supplementary appendix table S4).

#### Complications and AEs

Two participants in the exercise therapy group developed a frozen shoulder and one participant reported aggravation of low back pain over the course of exercise therapy regimen (table 3). No other AEs directly related to the exercise therapy were registered.

## Discussion

This multicentre, randomised, placebo controlled trial involving patients with shoulder impingement syndrome showed that ASD was not superior to diagnostic arthroscopy, with regard to any of the outcomes assessed at the end of a 5-year follow-up period. Although both groups had significant improvement in both primary outcomes, the patients assigned to ASD had no clinically relevant improvement over those assigned to diagnostic arthroscopy. In our secondary comparison, ASD was not superior to exercise therapy at 5 years.

### Comparison with other studies

There is no other randomised, placebo surgery controlled trial of the long-term efficacy of ASD. In the short-term (up to 2 years), ASD did not alleviate shoulder symptoms any more than placebo surgery.^12 21^ The existing long-term evidence of the effects of ASD, consisting of 5–10 years follow-up studies comparing ASD to exercise therapy,^7–10^ is conflicting. In three of these unblinded trials, there was no clinically meaningful benefit,^7 8 10^ while one trial suggested a benefit of ASD.^9^ The primary difference between our trial and other long-term follow-up studies is our placebo surgery controlled design. Acknowledging that the act of surgery per se produces a profound placebo response,^22–24^ a ‘true’ treatment effect is impossible to distinguish from the non-specific and placebo effects—such as the patients’ or researchers’ expectations of benefit—without a placebo comparison group.^25^ The only statistically significant between-group difference observed in our trial at 5 years was a 7-point benefit in the CM score, a secondary outcome, favouring ASD over diagnostic arthroscopy. However, the benefit observed in the CM score did not exceed either our pre-specified MID threshold of 17 points^18^ or the corresponding median MID estimate (8.3 points) published for this outcome in a recent systematic review.^4^ Given that we did not formally adjust for the number of secondary and exploratory outcomes, we argue that some positive findings among these eight outcomes, particularly when assessed in two parallel comparisons (ASD compared with both diagnostic arthroscopy and exercise therapy), might be expected on the basis of chance alone.

### Strengths and limitations of study

Our efficacy trial was designed to detect a treatment benefit of ASD. The surgeons and physiotherapists delivering care were highly experienced. We isolated subacromial decompression, the critical therapeutic element of the ASD procedure, as the only difference between ASD and diagnostic arthroscopy arms, while carefully maintaining all other care identically. We also enrolled only patients most likely to benefit from ASD. The efficacy design increases the generalisability of the findings: When no benefit is detected in an efficacy trial, there is no reason to believe that the tested intervention would be efficacious in a more heterogeneous population or under less than ideal circumstances, such as in a routine healthcare setting with less experienced surgeons.

Publication of the short-term results of this trial^12^ prompted criticism that our study was short term and underpowered.^26^ Failure to reach the prespecified target sample size might indeed be interpreted as evidence justifying such an assertion. However, once a study has been conducted, there is little merit in recalculating the statistical power as the credibility of the findings is appropriately indicated by confidence intervals.^27^ An increase in sample size simply yields smaller CIs while the effect estimates remain the same. Given that there were no between-group differences and the point estimates exclude clinically relevant treatment effects, our findings are not based on absence of evidence, as in an underpowered study, rather on evidence of absence of a clinically significant treatment effect. Also, had we simply used the more conventional 80% power—instead of the stringent 90% used in estimating sample size a priori—and maintained all other criteria constant, we would have met the recruitment target with the numbers we recruited.

Our decision to include pain at rest as one of our two primary outcomes can be criticised, as this outcome shows poor performance (responsiveness) and we did not set a threshold level of pain at rest (or activity) for patients to be included at entry. It follows that—theoretically—patients with low scores were eligible for the study and such patients would not have had the opportunity to improve. Although we agree that pain at rest indeed showed poor performance and we should have set a minimum pain threshold for eligibility for both primary outcomes, we respectfully remind the reader that our patients adequately represented the typical candidates for surgery due to ‘shoulder impingement’ as assessed by experienced shoulder surgeons. The patients included in our trial were all highly symptomatic at an average of 19 months after the onset of symptoms despite having undergone various treatments. Most importantly, there were no clinically relevant benefits of ASD in the other primary outcome (pain on activity), or any of the secondary outcomes.

Failure of the group differences to reach an MID threshold may hide major differences in the proportion of patients who attain an MID improvement. Thus, it may be argued that we should have reported the number of subjects in each treatment group who attained MID (ie, the participants who noticed at least some improvement). However, in patients with musculoskeletal conditions, feeling good rather than feeling better matters more),^28^ and we argue that this tenet is particularly pertinent when evaluating a surgical procedure. Therefore, rather than assessing the proportion of patients ‘feeling better’, we chose to carry out a ‘responder analysis’ in which we assessed the proportion of patients satisfied (‘feeling good’) with their treatment outcome. We found no difference between the three groups in the proportion of responders (tables 2 and 3).

Finally, a higher frequency of crossovers has been used as an argument to assert the superiority of surgery.^26^ Overall, we consider that the observed 5 years incidence of treatment conversions from diagnostic arthroscopy to ASD due to persisting symptoms (9/55, 16%) was low. Given that the decision to (re)operate is always made after unblinding of the treatment group allocation while the decision to unblind the treatment group is made without knowledge of the treatment given to the patient, we consider the frequency of “unblindings” a less biased measure of the severity of participants’ symptoms than the frequency of crossovers/reoperations. In our trial, we found no statistically significant difference in the frequency of unblindings between the ASD and diagnostic arthroscopy groups (6/59 in the ASD group vs 12/63 in the diagnostic arthroscopy group; p=0.25). The results of the primary comparison also remained unaltered in the prespecified sensitivity (as treated and per protocol) analyses.

As thoroughly elaborated previously,^12^ the interpretation of the findings of our pragmatic, exploratory secondary comparison between surgical and non-operative care (ASD vs exercise therapy) requires caution for at least two reasons: (1) it is not a blinded comparison, (2) because of the exclusions carried out in the group primarily allocated to surgery before the second randomisation, there is a clear prognostic imbalance that favours ASD, (3) the progressive exercise therapy regimen carried out in the exercise therapy group is different from the postoperative rehabilitation carried out by patients in the ASD group, as surgically treated patients need time to recover from the initial surgical trauma while also being subject to some degree of postoperative immobilisation, extended sick leave, and modifications in pain medication and activities. Despite this obvious bias, we failed to find any clinically relevant difference between the ASD and exercise therapy groups at the 5 years follow-up, a finding in agreement with the majority of previous unblinded trials comparing ASD to non-operative treatment.^7 8 10^

Given that recent trials questioning the viability of surgery for patients with subacromial pain syndrome have prompted lively debates, it seems that a wider perspective on this issue can be drawn from our data. All three groups in our trial achieved mean pain scores less than 20 out of 100 in the longer term. Although it may take those allocated to exercise a bit more time to achieve these low levels of pain, the effect does not seem to be explained by a very large number of participants crossing over, one could see this contributes to the argument of the conditions not requiring surgical intervention in the large majority of patients with subacromial pain.

## Conclusions and policy implications

The results of this randomised, placebo surgery controlled trial show that ASD provided no clinically relevant medium to long-term benefit over diagnostic arthroscopy in patients with shoulder impingement syndrome. The findings contest the current practice of performing subacromial decompression in patients with shoulder impingement syndrome and lend further support to existing guidelines that make a strong recommendation against surgery as a treatment for patients with subacromial pain. As the current evidence indicates that the impingement theory has become antiquated, we would also recommend to abandon the term shoulder impingement as it refers to this mechanical theory. The more generic term ‘subacromial pain’ should be preferred.

**What are the findings?**

- Our FIMPACT trial is the only placebo surgery controlled trial to assess the long-term (5 years) outcome of arthroscopic subacromial decompression (ASD) in patients with symptoms consistent with shoulder impingement syndrome.
- ASD and diagnostic arthroscopy (placebo surgery) (as well as exercise therapy) resulted in significant improvements in pain and functional outcomes with no difference in the incidence of adverse events.
- Patients assigned to ASD had no superior improvement over those assigned to diagnostic arthroscopy (or exercise therapy) at the 5 years follow-up.

**How might it impact on clinical practice in the future?**

- The findings contest the current practice of performing subacromial decompression in patients with shoulder impingement syndrome and lend further support to existing guidelines that make a strong recommendation against this surgery as a treatment for patients with subacromial pain.
