# Supplementary material for: Subacromial decompression versus diagnostic arthroscopy for shoulder impingement: a 5-year follow-up of a randomised, placebo surgery controlled clinical trial
Source: Br J Sports Med. 2020 Oct 5;55(2):99–107. doi: 10.1136/bjsports-2020-102216 (PMC7788208; doi:10.1136/bjsports-2020-102216)
Supplement: Supplementary data [file bjsports-2020-102216supp001.pdf]

Supplementary Appendix

This appendix has been provided by the authors to give readers additional information about their work.

Table of Contents

FIMPACT Investigators 3
Table S1 Trajectories of the primary outcomes at 3, 6, 12, 24 months and 5 years (ASD vs. DA) 5
Table S2 Trajectories of the secondary outcomes at 3, 6, 12, 24 months and 5 years (ASD vs. DA) 6
Table S3 Sensitivity analyses of the primary and secondary outcomes concerning the primary comparison (ASD vs. DA) 8
Table S4 Unblindings, treatment conversions, and reoperations 9
Table S5 Trajectories of the primary outcomes at 3, 6, 12, 24 months and 5 years (ASD vs. ET) 10
Table S6 Trajectories of the secondary outcomes at 3, 6, 12, 24 months and 5 years (ASD vs. ET) 11
Table S7 Sensitivity analyses of the primary and secondary outcomes concerning the secondary comparison (ASD vs. ET) 13
Table S8 Frequency of missing data at 3, 6, 12, 24 months and 5 years 14
Exercise Therapy (ET) Protocol 15

FIMPACT Investigators

Authors:

Mika Paavola, MD, PhD[1]; Kari Kanto, MD[2,3]; Jonas Ranstam, PhD[4]; Antti Malmivaara, MD, PhD[5]; Jari Inkinen, PT[6]; Juha Kalske, MD[7]; Vesa Savolainen, MD, PhD[8]; Ilkka Sinisaari, MD, PhD[9]; Simo Taimela, MD, PhD[1,2]; Teppo L N Järvinen, MD, PhD[1,2]

Collaborators:

Jarkko Pajarinen, MD, PhD[1]; Sikri Tukiainen, MD[10]; Kalevi Hietaniemi, MD, PhD[1]; Vesa Lepola, MD, PhD[11]; Jyrki Salmenkivi, MD[1]; Mikko Salmela, MD, PhD[1]; Timo Järvelä, MD, PhD[12]; Janne Lehtinen, MD, PhD[3]; Ville Haapamäki, MD, PhD[13]; Heikki Kolehmainen, MD, PhD[14]; Mathias Bäck, MD[1]; Mikael Salmela, MD[12]; Tarja Kunnala, PT[7]; Sami Niskanen, PT[8]; Hanna-Mari Laiho, PT[3]; Leena Kangas-Viri, PT[3]; Leena Caravitis, PT[1, 2]; Sanna Hokkanen RN[1]; Sari Karesvuori, RN[7]; Soile Lindholm, RN[1]; Pirjo Toivonen, PT[2]; Eero Hölli, PT[8]; Lena Laine, PT[10] and Esa Läärä, Professor[13]

[1] Department of Orthopedics and Traumatology, Helsinki University Hospital, Töölö hospital, Helsinki, Finland

[2] Finnish Centre for Evidence-Based Orthopedics (FICEBO), University of Helsinki, Helsinki, Finland

[3] Department of Orthopedics and Traumatology, Tampere University Hospital, TAYS Hatanpää, Tampere, Finland

[4] Mdas AB, Ystad, Sweden

[5] National Institute for Health and Welfare, Centre for Health and Social Economics, Helsinki, Finland

[6] Fysios Finlayson, Physiotherapy centre Fysios Finlayson Oy, Tampere, Finland

[7] Department of Orthopedics and Traumatology, Helsinki University Hospital, Jorvi hospital, Espoo, Finland

[8] Pohjola Hospital, Helsinki, Finland

[9] Terveystalo, Helsinki, Finland

[10] Mehiläinen, Helsinki, Finland

[11] Pohjola Hospital, Tampere, Finland

[12] Mehiläinen, Tampere, Finland

[13] HUS Medical Imaging Centre, Helsinki, Finland

[14] Department of Radiology, Tampere University Hospital, TAYS Hatanpää, Tampere, Finland

[16] Research Unit of Mathematical Sciences, University of Oulu, Oulu, Finland

Contributions:

Writing Committee: MP, KK, ST, and TJ.

Steering Committee: MP, ST, AM, and TJ.

Clinical Sites:

Helsinki University Hospital, Jorvi Hospital, Helsinki:

Clinical Site Investigators: Kalevi Hietaniemi, Juha Kalske, Vesa Lepola, Jyrki Salmenkivi and Sikri Tukiainen.

Physiotherapy (follow-up examinations): Tarja Kunnala

Imaging: Ville Haapamäki

Research coordinators: Leena Caravitis, Sari Karesvuori

Helsinki University Hospital, Herttoniemi Hospital, Helsinki:

Clinical Site Investigators: Jarkko Pajarinen, Mikko Salmela, Vesa Savolainen and Ilkka Sinisaari

Physiotherapy (follow-up examinations): Sami Niskanen

Research coordinators: Leena Caravitis and Soile Lindholm

Tampere University Hospital, TAYS Hatanpää, Tampere:

Clinical Site Investigators: Timo Järvelä, Kari Kanto, Janne Lehtinen and Mikael Salmela

Imaging: Heikki Kolehmainen

Physiotherapy (follow-up examinations): Hanna-Mari Laiho and Leena Kangas-Viri

Research coordinator: Pirjo Toivonen.

Independent Physiotherapists:

Jari Inkinen, Eero Hölli, Lena Laine

FIMPACT Methods Center:

Pirjo Toivonen (Project management), Mathias Bäck (Data management) and Esa Läärä (Randomisation).

Table S1 Trajectories of the primary outcomes at 3, 6, 12, 24 months and 5 years (ASD vs. DA)
The table shows the mean values and between-group differences in VAS pain scores at the 3, 6, 12, 24-month and 5-year follow-ups with 95% confidence intervals. The trajectories are estimated using a mixed-effects model repeated measures (MMRM) ANOVA with adjustment for baseline imbalance. N denotes the number of subjects.

Outcomes	Arthroscopic subacromial decompression (ASD)	n	Diagnostic arthroscopy (DA)	n	Between-group difference, ASD vs. DA
**3 months**					
Pain VAS at rest	21.6 (17.0 to 26.3)	54	19.8 (15.0 2o 24.3)	55	1.9 (-4.6 to 8.4)
Pain VAS on arm activity	42.1 (35.4 to 48.8)	52	37.5 (31.0 to 44.0)	55	4.6 (-4.8 to 14.0)
**6 months**					
Pain VAS at rest	15.9 (11.3 to 20.5)	59	13.9 (9.4 to 18.5)	61	1.9 (-4.3 to 8.2)
Pain VAS on arm activity	37.9 (31.5 to 44.4)	59	37.7 (31.5 to 43.9)	61	0.1 (-8.7 to 9.0)
**12 months**					
Pain VAS at rest	11.6 (7.0 to 16.2)	55	13.9 (9.6 to 18.3)	56	-1.5 (-8.0 to 4.9)
Pain VAS on arm activity	23.3 (16.7 to 29.8)	55	28.0 (21.5 to 34.4)	56	-4.7 (-13.8 to 4.5)
**24 months**					
Pain VAS at rest	5.2 (0.8 to 9.6)	59	9.8 (5.2 to 14.2)	59	-4.6 (-10.8 to 1.7)
Pain VAS on arm activity	15.8 (9.5 to 22.1)	59	24.8 (18.5 to 31.1)	59	-9.0 (-17.9 to 0)
**5 years**					
Pain VAS at rest	6.2 (1.6 to 10.8)	54	8.2 (3.5 to 12.8)	54	-2.0 (-8.5 to 4.6)
Pain VAS on arm activity	12.4 (5.8 to 19.0)	54	20.4 (13.8 to 26.9)	54	-8.0 (-17.3 to 1.3)

The between-group differences may not exactly equal the differences in change in the scores between the ASD and DA groups because of the adjustment for baseline imbalance in the MMRM analyses. A negative between-group difference means that the participants in the ASD group reported a lower pain level.

The minor discrepancies in the results of the ASD group in this Table S2 and in the Table S6 are due to the adjustment for baseline imbalance when analysing change from baseline. The adjustment differs based on which groups are included in the model.

Table S2 Trajectories of the secondary outcomes at 3, 6, 12, 24 months and 5 years (ASD vs. DA)
The table shows the mean values and between-group differences in VAS pain scores at the 3, 6, 12 and 24-month and 5-year follow-ups with 95% confidence intervals. The trajectories are estimated using a mixed-effects model repeated measures (MMRM) ANOVA with adjustment for baseline imbalance. N denotes the number of subjects.

Outcomes	Arthroscopic subacromial decompression (ASD)	n	Diagnostic arthroscopy (DA)	n	Between-group difference ASD vs. DA
**3 months**					
Constant-Murley score	N/A		N/A		N/A
SST	N/A		N/A		N/A
Satisfaction to the treatment (0-100 VAS)	76.9 (71.9 to 82.0)	57	81.4 (76.3 to 86.4)	57	-4.5 (-11.6 to 2.7)
15-D	0.91 (0.90 to 0.92)	57	0.92 (0.91 to 0.93)	57	-0.01 (-0.02 to 0.01)
SF-36 score Physical health	81.6 (78.3 to 84.8)	58	83.2 (79.9 to 86.5)	57	-1.6 (-6.2 to 3.0)
Mental health	79.8 (77.0 to 82.7)	58	83.2 (80.3 to 86.1)	57	-3.3 (-7.4 to 0.8)
**6 months**					
Constant-Murley score	58.2 (54.0 to 62.4)	59	64.4 (60.2 to 68.5)	61	-6.1 (-12.1 to 0.2)
SST	7.9 (7.4 to 8.5)	59	8.5 (7.9 to 9.1)	61	-0.5 (-1.4 to 0.3)
Satisfaction to the treatment (0-100 VAS)	75.1 (70.1 to 80.2)	56	79.7 (74.7 to 84.7)	58	-4.5 (-11.7 to 2.6)
15-D	0.92 (0.90 to 0.93)	56	0.92 (0.91 to 0.94)	58	-0.01 (-0.02 to 0.01)
SF-36 score Physical health	84.9 (81.6 to 88.1)	57	84.7 (81.5 to 88.0)	58	0.1 (-4.5 to 4.8)
Mental health	80.6 (77.7 to 83.5)	57	82.3 (79.4 to 85.1)	59	-1.6 (-5.7 to 2.5)
**12 months**					
Constant-Murley score	N/A		N/A		N/A
SST	N/A		N/A		N/A
Satisfaction to the treatment (0-100 VAS)	82.9 (77.8 to 88.0)	55	85.9 (80.7 to 91.0)	54	-2.9 (-10.2 to 4.3)
15-D	0.92 (0.91 to 0.93)	56	0.93 (0.92 to 0.94)	55	-0.01 (-0.03 to 0)
SF-36 score Physical health	86.9 (83.6 to 90.1)	56	85.3 (82.0 to 88.6)	56	1.5 (-3.2 to 6.2)
Mental health	77.0 (74.1 to 80.0)	55	85.2 (82.2 to 88.2)	57	-8.2 (-12.4 to 4.0)
**24 months**					
Constant-Murley score	78.0 (73.7 to 82.3)	58	73.8 (69.6 to 78.0)	59	4.2 (-1.8 to 10.2)
SST	10.4 (9.8 to 11.0)	58	9.9 (9.3 to 10.5)	59	0.4 (-0.4 to 1.3)
Satisfaction to the treatment (0-100 VAS)	88.2 (83.2 to 93.3)	58	87.4 (82.4 to 92.5)	56	0.8 (-6.3 to 7.9)
15-D	0.92 (0.91 to 0.93)	58	0.92 (0.91 to 0.94)	58	-0.0 (-0.02 to 0.01)

SF-36 score Physical health	87.6 (84.4 to 90.9)	58	86.1 (82.9 to 89.4)	58	1.5 (-3.1 to 6.1)
Mental health	77.9 (75.0 to 80.8)	57	82.6 (79.7 to 85.5)	59	-4.7 (-8.8 to 0.6)
**5 years**					
Constant-Murley score	82.8 (78.4 to 87.3)	58	75.7 (71.3 to 80.1)	59	7.1 (-0.9 to 13.4)
SST	10.7 (10.1 to 11.3)	58	10.3 (9.7 to 11.0)	59	0.3 (-0.5 to 1.2)
Satisfaction to the treatment (0-100 VAS)	89.7 (84.5 to 94.9)	58	85.7 (80.6 to 90.8)	56	4.0 (-3.2 to 11.3)
15-D	0.92 (0.90 to 0.92)	58	0.92 (0.90 to 0.93)	58	-0.01 (-0.03 to 0.01)
SF-36 score Physical health	84.0 (80.6 to 87.4)	58	85.4 (82.0 to 88.8)	57	-1.4 (-6.1 to 3.4)
Mental health	79.9 (76.9 to 82.9)	57	80.1 (77.0 to 83.2)	57	-0.2 (-4.5 to 4.1)

The between-group differences may not exactly equal the differences in change in the scores between the ASD and DA groups because of the adjustment for baseline imbalance in the MMRM analyses. N/A = not applicable. For all variables, a higher score indicates a better treatment outcome.

Table S3 Sensitivity analyses of the primary and secondary outcomes concerning the primary comparison (ASD vs. DA)

The table shows the between-group differences at the 5-year follow up. The estimated effect indicates the mean difference, ASD minus DA. The analyses were carried out using a mixed model repeated measures (MMRM) ANOVA with adjustment for baseline imbalance.

Variable	ITT Estimated effect (95% CI)*	P value	Per protocol Estimated effect (95% CI)†	P value	As treated Estimated effect (95% CI)‡	P value
Pain VAS at rest	-2.0 (-8.5 to 4.6)	0.56	-4.0 (-10.5 to 2.5)	0.23	-5.9 (-12.4 to 0.6)	0.077
Pain VAS at activity	-8.0 (-17.3 to 1.3)	0.092	-9.9 (-19.4 to -0.2)	0.046	-9.5 (-18.6 to -0.3)	0.043
Constant-Murley score	7.1 (0.9 to 13.4)	0.025	7.5 (1.1 to 13.8)	0.021	6.3 (0.1 to 12.5)	0.046
SST	0.3 (-0.5 to 1.2)	0.45	0.4 (-0.5 to 1.3)	0.37	0.6 (-0.3 to 1.4)	0.20
Satisfaction to the treatment (VAS 0-100)	4.0 (-3.2 to 11.3)	0.28	5.6 (-1.9 to 13.1)	0.14	5.1 (-2.2 to 12.4)	0.17
15-D	-0.01 (-0.03 to 0.01)	0.32	-0.01 (-0.03 to 0.01)	0.32	-0.01 (-0.03 to 0.01)	0.32
SF-36 score Physical health	-1.4 (-6.1 to 3.4)	0.58	-0.9 (-5.8 to 4.1)	0.73	-0.1 (-4.8 to 4.6)	0.97
Mental health	-0.2 (-4.5 to 4.1)	0.92	0.5 (-3.9 to 5.1)	0.81	0.8 (-3.5 to 5.1)	0.71

* ITT, the intention to treat population (ASD: n = 59, DA: n = 63).

† The per protocol population is the subset of the intention to treat population who received the treatment they were randomised to and who did not receive any other treatment, i.e. the patients with a treatment conversion have been excluded (ASD: n = 59, DA: n = 54).

‡ The as treated population is defined according to the treatment the participants received, i.e. the 9 participants who originally received DA and the 14 participants who originally received ET, but due to persistent symptoms requested unblinding and subsequently received ASD, have been included in the ASD population (ASD: n = 83, DA: n = 54).

Table S4 Unblindings, treatment conversions, and reoperations				
**Study group**	**Unblinding or decision on treatment conversion. Time from randomisation (Months)**	**Treatment converted to***	**Reoperations**	**Time from randomisation to reoperation (months)**
ET	2	ASD		
ET	5	ASD	MUA and ADCR	10
ET	6	ASD and MUA		
ET	7	ASD		
ET	7	ASD		
ET	7	ASD	ASD and LHBT	23
ET	7	ASD		
ET	7	ASD		
ET	8	ASD		
ET	8	ASD		
ET	9	ASD	ASD and ADCR	57
ET	11	ASD		
ET	12	ASD		
ET	15	ADCR		
ET	21	ACR and ASD		
ET	55	ASD		
ASD	3			
ASD	8			
ASD	8	MUA		
ASD	13			
ASD	14			
ASD	18	ADCR	ASD and ADCR	51
DA	1	ASD		
DA	5	ASD		
DA	6			
DA	9	ASD		
DA	9	ASD		
DA	9	ASD		
DA	11	ASD		
DA	12	ASD		
DA	19	ASD and SSC repair		
DA	25			
DA	26	ASD and DPC		
DA	55	SSP repair (trauma)		

Abbreviations: ASD=Arthroscopic Subacromial Decompression; ACR=Arthroscopic Capsular Release; MUA=Manipulation Under Anesthesia; ADCR=Arthroscopic Distal Clavicle Resection; LHBT=Long Head of the Biceps Tendon; SSC repair=Subscapularis tendon repair; SSP repair=Supraspinatus tendon repair; DPC= Drainage of a paralabral cyst.

*Treatment conversions (surgeries) were carried out within 4 months of unblinding (mean: 6 weeks, range 1-16 weeks).

Table S5 Trajectories of the primary outcomes at 3, 6, 12, 24 months and 5 years (ASD vs. ET)
The table shows the mean values and between-group differences in VAS pain scores at the 3, 6, 12, 24-month and 5-year follow-ups with 95% confidence intervals. The trajectories are estimated using a mixed-effects model repeated measures (MMRM) ANOVA with adjustment for baseline imbalance. N denotes the number of subjects.

Outcomes	Arthroscopic subacromial decompression (ASD)	n	Exercise Therapy (ET)	n	Between-group difference, ASD vs. ET
**3 months**					
Pain VAS at rest	21.7 (17.0 to 26.5)	54	23.8 (19.4 to 28.3)	62	-2.1 (-8.7 to 4.4)
Pain VAS on arm activity	42.3 (35.7 to 49.0)	52	44.7 (38.7 to 50.8)	62	-2.4 (-11.5 to 7.0)
**6 months**					
Pain VAS at rest	15.9 (11.4 to 20.5)	59	16.9 (12.6 to 21.2)	68	-0.9 (-7.2 to 5.4)
Pain VAS on arm activity	38.1 (31.8 to 44.4)	59	44.6 (38.7 to 50.5)	68	-6.5 (-15.1 to 2.1)
**12 months**					
Pain VAS at rest	11.6 (6.9 to 16.4)	55	17.1 (12.8 to 21.5)	65	-5.5 (-11.9 to 0.9)
Pain VAS on arm activity	23.5 (17.0 to 30.0)	55	33.1 (27.1 to 39.1)	64	-9.6 (-18.4 to -0.8)
**24 months**					
Pain VAS at rest	5.3 (0.7 to 9.9)	59	12.8 (8.5 to 17.1)	68	-7.5 (-13.8 to -1.2)
Pain VAS on arm activity	16.0 (9.7 to 22.3)	59	28.1 (22.2 to 34.0)	68	-12.1 (-20.7 to -3.5)
**5 years**					
Pain VAS at rest	6.3 (1.5 to 11.0)	59	5.3 (0.8 to 9.8)	68	-1.0 (-5.6 to 7.6)
Pain VAS on arm activity	12.6 (6.1 to 19.2)	59	16.5 (10.3 to 22.6)	68	-3.9 (-12.8 to 5.1)

The between-group differences may not exactly equal the differences in change in the scores between the ASD and DA groups because of the adjustment for baseline imbalance in the MMRM analyses. A negative between-group difference means that the participants in the ASD group reported a lower pain level.

The minor discrepancies in the results of the ASD group in this Table S6 and in the Table S2 are due to the adjustment for baseline imbalance when analysing change from baseline. The adjustment differs based on which groups are included in the model.

Table S6 Trajectories of the secondary outcomes at 3, 6, 12, 24 months and 5 years (ASD vs. ET)

The table shows the mean values and between-group differences in VAS pain scores at the 3, 6, 12 ,24-month and 5-year follow-ups with 95% confidence intervals. The trajectories are estimated using a mixed-effects model repeated measures (MMRM) ANOVA with adjustment for baseline imbalance. N denotes the number of subjects.

Outcomes	Arthroscopic subacromial decompression (ASD)	n	Exercise Therapy (ET)	n	Between-group difference ASD vs. ET
**3 months**					
Constant-Murley score	N/A		N/A		N/A
SST	N/A		N/A		N/A
Satisfaction to the treatment (0-100 VAS)	76.9 (71.8 to 82.0)	57	79.3 (74.5 to 84.0)	67	-2.3 (-9.3 to 4.6)
15-D	0.91 (0.89 to 0.92)	57	0.90 (0.89 to 0.91)	65	0.01 (-0.01 to 0.30)
SF-36 score Physical health	81.8 (78.9 to 84.8)	58	81.3 (78.5 to 84.0)	66	0.6 (-3.5 to 4.6)
Mental health	79.1 (76.0 to 82.2)	58	80.2 (77.3 to 83.2)	67	-1.1 (-5.4 to 3.2)
**6 months**					
Constant-Murley score	59.1 (54.8 to 63.4)	59	58.3 (54.3 to 62.3)	68	0.8 (-5.0 to 6.7)
SST	7.9 (7.3 to 8.5)	59	7.9 (7.4 to 8.5)	68	0.0 (-0.80 to 0.80)
Satisfaction to the treatment (0-100 VAS)	75.2 (67.0 to 80.3)	56	75.2 (70.4 to 80.0)	66	-0.0 (-7.1 to 7.0)
15-D	0.91 (0.90 to 0.93)	56	0.89 (0.88 to 0.90)	66	0.02 (0 to 0.04)
SF-36 score Physical health	85.1 (82.1 to 88.1)	57	81.3 (78.6 to 84.1)	67	3.8 (-0.2 to 7.8)
Mental health	79.9 (76.7 to 83.0)	57	80.4 (77.4 to 83.3)	67	-0.5 (-4.8 to 3.8)
**12 months**					
Constant-Murley score	N/A		N/A		N/A
SST	N/A		N/A		N/A
Satisfaction to the treatment (0-100 VAS)	82.9 (77.7 to 88.1)	55	78.0 (73.2 to 82.9)	64	4.9 (-2.2 to 12.0)
15-D	0.91 (0.90 to 0.93)	56	0.91 (0.89 to 0.92)	64	0.01 (-0.01 to 0.03)
SF-36 score Physical health	87.1 (84.1 to 90.1)	56	86.5 (83.6 to 89.3)	64	0.6 (-3.5 to 4.7)
Mental health	76.3 (73.1 to 79.5)	55	81.8 (78.7 to 84.8)	64	-5.5 (-9.9 to 1.1)
**24 months**					
Constant-Murley score	78.9 (74.6 to 83.2)	58	71.3 (67.3 to 75.4)	65	7.5 (1.6 to 13.4)
SST	10.3 (9.7 to 10.9)	58	9.7 (9.1 to 10.2)	66	0.7 (-0.1 to 1.5)
Satisfaction to the treatment (0-100 VAS)	88.2 (83.1 to 93.4)	58	84.8 (80.1 to 89.6)	67	3.4 (-3.5 to 10.4)
15-D	0.92 (0.90 to 0.93)	56	0.91 (0.90 to 0.92)	68	0.00 (-0.01 to 0.02)
SF-36 score Physical health	87.9 (85.0 to 90.8)	58	87.3 (84.5 to 90.0)	68	0.6 (-3.4 to 4.6)
Mental health	77.2 (74.0 to 80.3)	57	82.8 (79.8 to 85.7)	68	-5.6 (-9.9 to -1.3)

**5 years**					
Constant-Murley score	83.7 (79.2 to 88.1)	58	79.8 (75.6 to 88.1)	65	3.9 (2.2 to 10.0)
SST	10.6 (10.0 to 11.3)	58	10.7 (10.1 to 11.2)	66	0.0 (-0.9 to 0.8)
Satisfaction to the treatment (0-100 VAS)	89.7 (84.4 to 95.0)	58	86.8 (81.8 to 91.7)	67	3.0 (-4.3 to 10.2)
15-D	0.90 (0.89 to 0.91)	56	0.91 (0.90 to 0.93)	68	-0.01 (-0.03 to 0.01)
SF-36 score Physical health	84.3 (81.2 to 87.3)	52	87.5 (84.7 to 90.4)	62	-3.3 (-7.4 to 0.9)
Mental health	79.2 (75.9 to 82.4)	52	81.9 (78.8 to 84.9)	62	-2.7 (-7.2 to 1.8)

The between-group differences may not exactly equal the differences in change in the scores between the ASD and ET groups because of the adjustment for baseline imbalance in the MMRM analyses. N/A = not applicable. For all variables, a higher score indicates a better treatment outcome.

Table S7 Sensitivity analyses of the primary and secondary outcomes concerning the secondary comparison (ASD vs. ET)
The table shows the between-group differences at the 5-year follow-up. The estimated effect indicates the mean difference, ASD minus ET.
The analyses were carried out using a mixed model repeated measures (MMRM) ANOVA with adjustment for baseline imbalance.

Variable	FAS Estimated effect (95% CI)*	P value	Per protocol Estimated effect (95% CI)†	P value	As treated Estimated effect (95% CI)‡	P value
Pain VAS at rest	1.0 (-5.6 to 7.6)	0.77	0.2 (-6.5 to 6.9)	0.96	-1.5 (-8.2 to 5.1)	0.65
Pain VAS at activity	-3.9 (-12.8 to 5.1)	0.40	-3.6 (-12.9 to 5.7)	0.45	-3.2 (-12.1 to 5.7)	0.48
Constant-Murley score	3.9 (-2.3 to 10.0)	0.22	3.5 (-2.8 to 10.7)	0.28	2.7 (-3.5 to 8.8)	0.40
SST	-0 (-0.8 to 0.9)	0.70	0.2 (-0.7 to 1.0)	0.70	0.3 (-0.5 to 1.1)	0.43
Satisfaction to treatment (0-100 VAS)	3.0 (-4.3 to 10.2)	0.42	2.0 (-5.2 to 9.3)	0.58	1.5 (-5.6 to 8.7)	0.67
15-D	-0.01 (-0.03 to 0.01)	0.32	-0.02 (-0.03 to -0)	0.046	-0.02 (-0.03 to 0)	0.046
SF-36 score Physical health	-3.3 (-7.4 to 0.9)	0.13	-3.8 (-8.1 to 0.4)	0.074	-2.9 (-7.1 to 1.3)	0.17
Mental health	-2.7 (-7.2 to 1.8)	0.23	-4.0 (-8.7 to 0.6)	0.089	-3.81 (-8.2 to 0.6)	0.089

* FAS, the full analysis set population (ASD: n = 59, ET: n = 71).

† The per protocol population is the subset of the full analysis set population who received the treatment they were randomised to and who did not receive any other treatment, i.e. the patients with a treatment conversion have been excluded (ASD: n = 59, ET: n = 56).

‡ The as treated population is defined according to the treatment the participants received, i.e., the 9 participants who originally received DA and the 15 participants who originally received ET, but due to persistent symptoms requested unblinding and subsequently received ASD, have been included in the ASD population (ASD: n = 83, ET: n = 56).

Table S8 Frequency of missing data at 3, 6, 12, 24 months and 5 years

Study group	Timepoint	Pain VAS at rest	Pain VAS at activity	Constant-Murley score	SST	15D
ASD (n=59)	Baseline	0	0	0	0	2
	3 months	5	7	N/A	N/A	2
	6 months	0	0	0	0	3
	12 months	4	4	N/A	N/A	2
	24 months	0	0	1	1	1
	5 years	5	5	5	7	6
DA (n=63)	Baseline	0	0	0	0	3
	3 months	8	8	N/A	N/A	6
	6 months	2	2	2	2	5
	12 months	7	7	N/A	N/A	8
	24 months	4	4	4	4	5
	5 years	9	9	9	10	12
ET (n=71)	Baseline	1	1	1	1	2
	3 months	9	9	N/A	N/A	6
	6 months	3	3	3	3	5
	12 months	6	7	N/A	N/A	7
	24 months	3	3	6	5	3
	5 years	9	9	11	9	12

Abbreviations: ASD = Arthroscopic Subacromial Decompression; DA = Diagnostic arthroscopy; ET = Exercise therapy; VAS = Visual analogue scale; SST = Simple Shoulder Test.

The n values denote the number of participants allocated to each treatment group. The frequencies include both missing data and withdrawn subjects.

Exercise Therapy (ET) Protocol

**Phase I (weeks 0-3)**	
**Goals: Decrease pain, restore A/PROM, relieve pain and inflammation, re-establish muscular balance, and improve posture; Inform the patient**	
**Passive exercises:**<br>PROM<br>Joint mobilisation/posterior capsule stretching<br>Soft tissue mobilisation	**Active exercises:**<br>AAROM<br>Submaximal isometric rotator cuff exercises<br>Thoracic spine mobilisation<br>Scapular retraction/protraction
**Phase II (weeks 4-5)**	
**Goals: Re-establish full and pain free AROM, restore rotator cuff strength, restore normal scapulothoracic motion**	
**Passive exercises:**<br>Joint mobilisation/posterior capsule stretching<br>Soft tissue mobilisation	**Active exercises:**<br>AROM<br>Maximal isometric rotator cuff exercises<br>Thoracic spine mobilisation<br>Scapulothoracic motion
**Phase III (weeks 6-8)**	
**Goals: Restore muscle strength and endurance, re-establish full and pain free AROM. Restore normal scapulothoracic motion**	
**Passive exercises:**<br>Joint mobilisation	**Active exercises:**<br>Dynamic rotator cuff exercises<br>Scapulothoracic motion
**Phase IV (weeks 9-12)**	
**Goals: Enhance muscle strength and endurance, re-educate neuromuscular control of rotator cuff muscles**	
**Passive exercises:**<br>Continued stretching program	**Active exercises:**<br>Continued dynamic rotator cuff program

Abbreviations: PROM, Passive range of motion; AAROM, Active-assisted range of motion; AROM: Active range of motion

Exercise therapy (ET) group: home exercise program

Phase I (0-3 weeks)

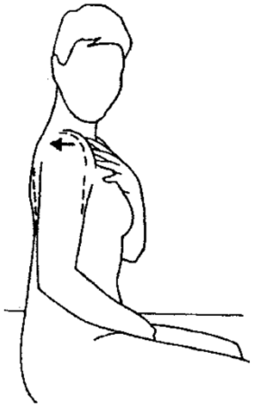

Sit. Place your hand on the front of the opposite shoulder. Feel your shoulder with your fingers making sure that the shoulder does not come forward. Move your shoulder gently 1 cm back and 2 cm up. Later on, exercise is done without palpation or visual confirmation of movement.

Repeat 3 x 15-25 times.

© PhysioTools Ltd

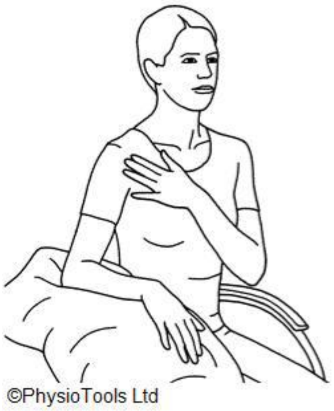

Sit. Place your hand on the front of the opposite shoulder. Feel your shoulder with your fingers. Move your shoulder gently 1 cm forward and 2 up from resting position. Later on, exercise is done without palpation or visual confirmation of movement.

Repeat 3 x 15-25 times.

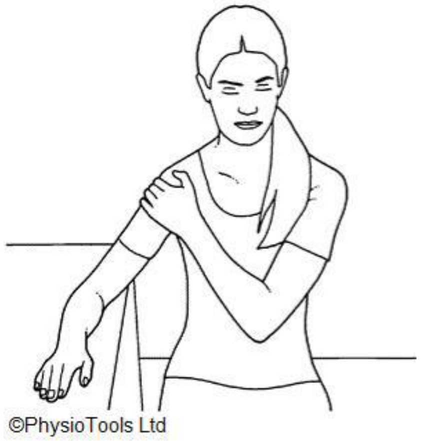

Sit on a chair with your arm supported on a table.
With your other hand push the top of your upper arm downwards.
Hold approx. 2 secs

Repeat 10 times.

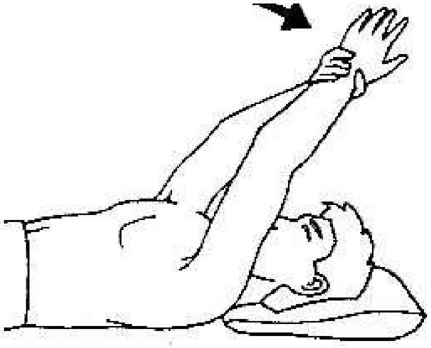

© PhysioTools Ltd

Lying on your back with elbows straight. Use one arm to lift the other arm up keeping it as close to the ear as possible.

Repeat 10 times.

BMJ Publishing Group Limited (BMJ) disclaims all liability and responsibility arising from any reliance placed on this supplemental material which has been supplied by the author(s)

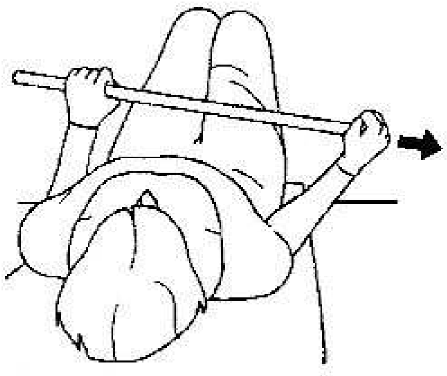

© PhysioTools Ltd

Lying on your back with elbows against your body and at a right angle. Hold a stick in your hands. Move the stick sideways thus pushing the arm to be exercised outwards.

Repeat 10 times.

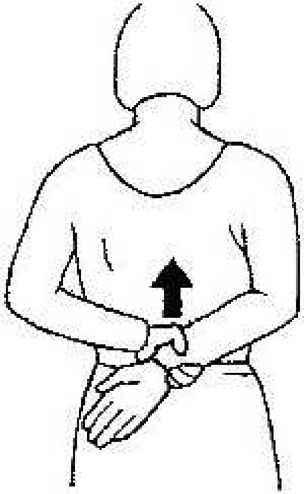

© PhysioTools Ltd

Stand with arms behind your back. Grasp the wrist of the arm you want to exercise. Slide your hands up the back.

Repeat 10 times.

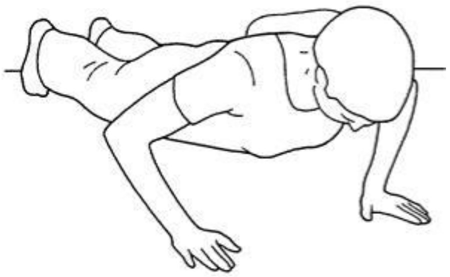

©PhysioTools Ltd

Lying face down with your hands on the floor at shoulder height. Do push-ups slowly and remember to straighten your elbows properly.

Repeat 3 x 15-25 times.

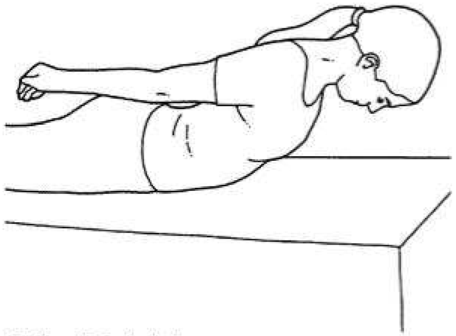

©PhysioTools Ltd

Lying face down, arms behind your back. Lift your upper trunk off the floor and pull your shoulder blades together. Look down at the floor while doing the exercise.

Repeat 3 x 15-25 times.

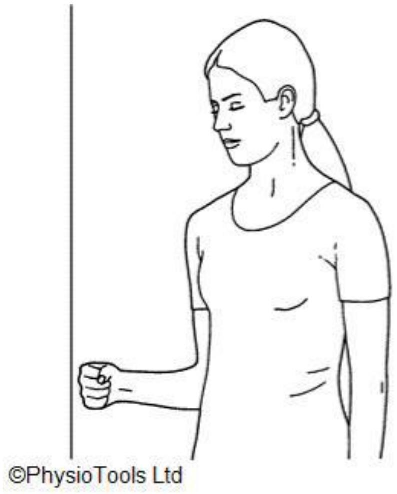

Stand sideways against a wall with your upper arm close to your side and elbow at a right angle. Push the forearm to the side against the wall. Hold approx. 5 secs.

Repeat 3 x 3 times.

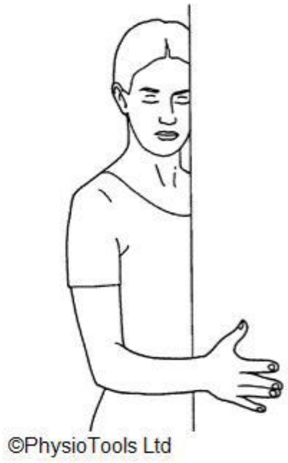

Stand in a doorway with you elbow close to your body and bent at a right angle. Place your hand against the wall. Push your hand inwards against the wall. Hold 5 secs. Relax.

Repeat 3 x 3 times.

BMJ Publishing Group Limited (BMJ) disclaims all liability and responsibility arising from any reliance placed on this supplemental material which has been supplied by the author(s)

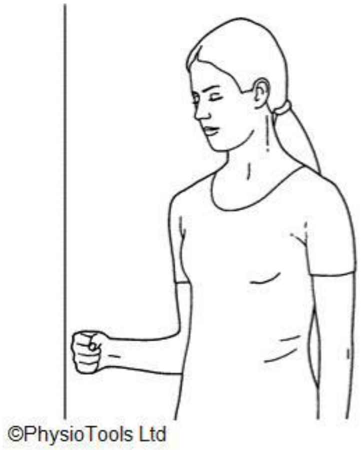

Stand with your upper arm close to your side, elbow at a right angle and the back of your hand against a wall. Push the back of your hand against the wall. Hold approx. 5secs.

Repeat 3 x 3times.

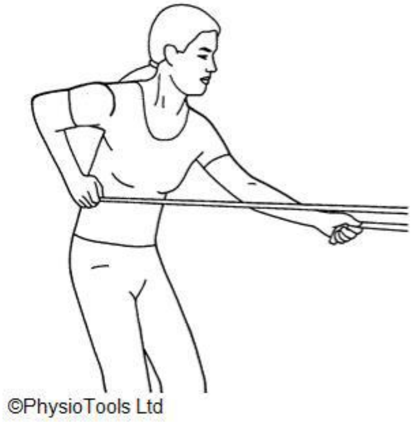

Stand facing a rubber exercise band with your knees and hips slightly bent. Pull the band alternately with the left and right hand keeping the pelvis still.

Repeat 3 x 15-25 times.

22

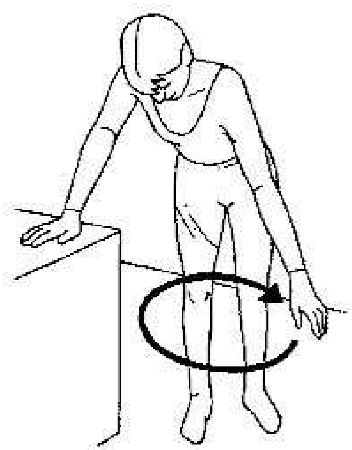

© PhysioTools Ltd

Stand leaning on a table with one hand. Let your other arm hang relaxed straight down. Swing your arm as if drawing a circle on the floor. Change direction.

Continue for 1-2 minutes

Phase II (4-5 weeks)

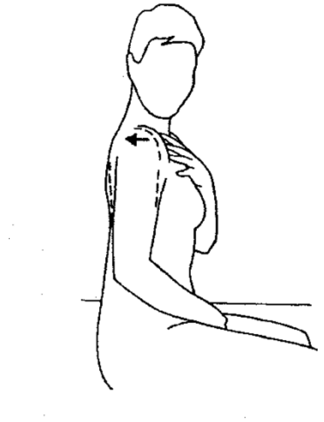

© PhysioTools Ltd

Sit. Place your hand on the front of the opposite shoulder. Feel your shoulder with your fingers making sure that the shoulder does not come forward. Move your shoulder gently 1 cm back and 2 cm up. Later on, exercise is done without palpation or visual confirmation of movement.

Repeat 3 x 15-25 times.

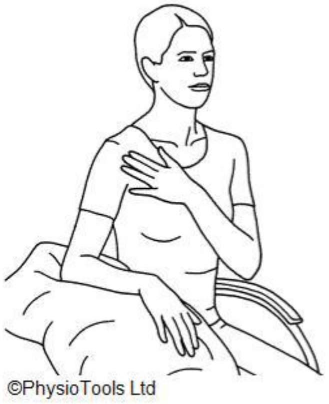

Sit. Place your hand on the front of the opposite shoulder. Feel your shoulder with your fingers. Move your shoulder gently 1 cm forward and 2 up from resting position. Later on, exercise is done without palpation or visual confirmation of movement.

Repeat 3 x 15-25 times.

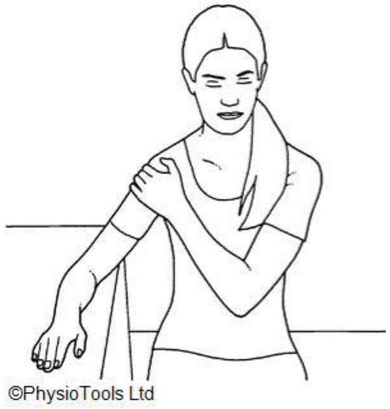

Sit on a chair with your arm supported on a table.
With your other hand push the top of your upper arm downwards. Hold approx. 2 secs.

Repeat 10 times.

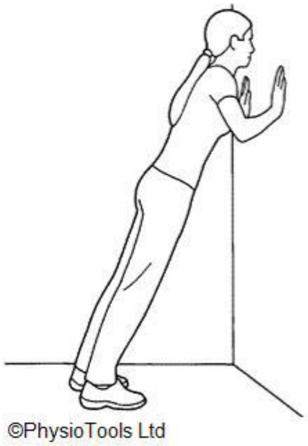

Stand facing a wall with your arms straight and hands on the wall. Do push-ups against the wall keeping your body in a straight line.

Repeat 3 x 15-25 times.

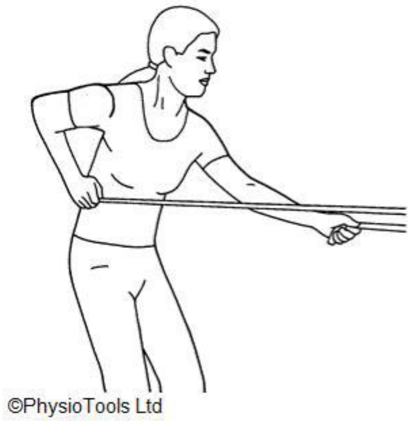

Stand facing a rubber exercise band with your knees and hips slightly bent. Pull the band alternately with the left and right hand keeping the pelvis still.

Repeat 3 x 15-25 times.

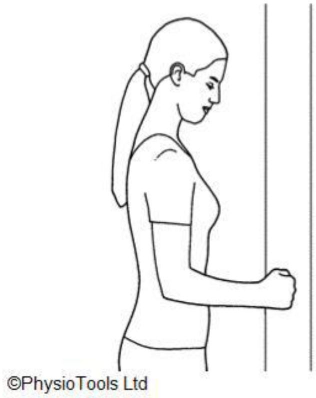

Stand facing a wall. Keep your upper arm close to the side with elbow at a right angle. Push your fist against the wall for 5 secs.

Repeat 3 x 3 times.

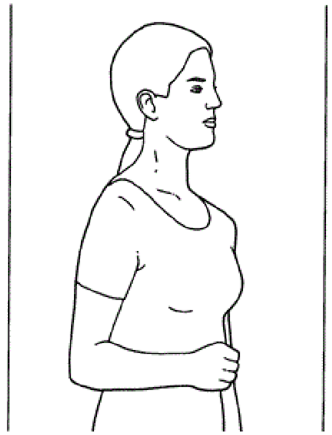

©PhysioTools Ltd

Stand with your back against the wall. Keep your upper arm close to the side and elbow at a right angle. Push the elbow back against the wall and hold for 5 secs.

Repeat 3 x 3 times.

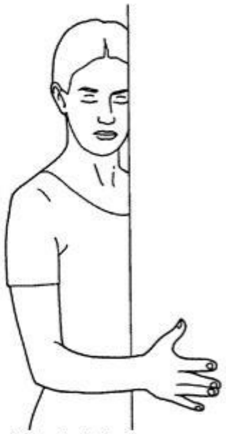

©PhysioTools Ltd

Stand in a doorway with you elbow close to your body and bent at a right angle. Place your hand against the wall. Push your hand inwards against the wall. Hold 5 secs.

Repeat 3 x 3 times.

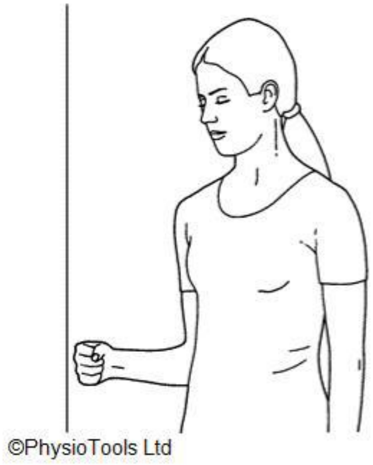

Stand with your upper arm close to your side, elbow at a right angle and the back of your hand against a wall. Push the back of your hand against the wall. Hold approx. 5 secs.

Repeat 3 x 3times.

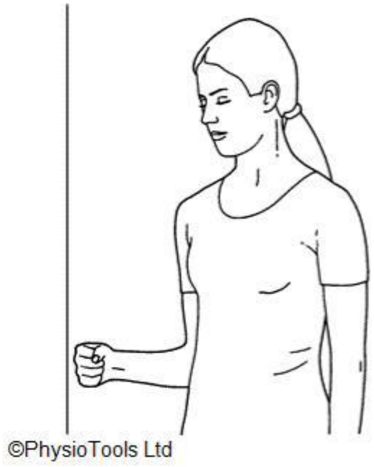

Stand sideways against a wall with your upper arm close to your side and elbow at a right angle. Push the forearm to the side against the wall. Hold approx. 5 secs.

Repeat 3 x 3 times.

28

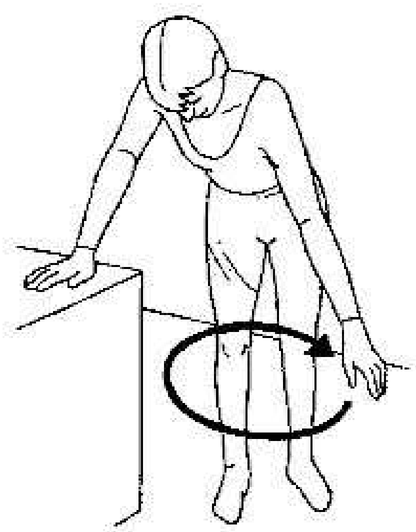

© PhysioTools Ltd

Stand leaning on a table with one hand. Let your other arm hang relaxed straight down. Swing your arm as if drawing a circle on the floor. Change direction.

Continue for 1-2 minutes

Phase III (6-8 weeks)

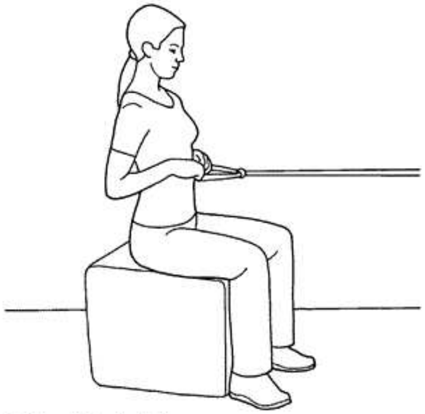
©PhysioTools Ltd

Sit or stand holding on to a rubber exercise band with both hands. Pull the band with both arms pushing the shoulder blades together.

Repeat 3 x 15-25 times.

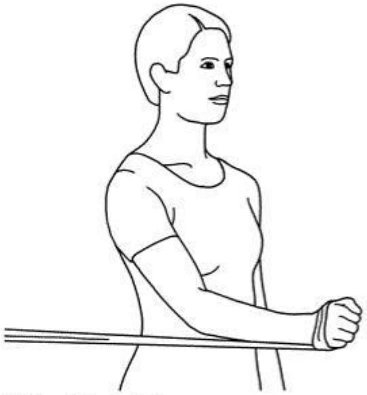
©PhysioTools Ltd

Stand straight holding an exercise band. Move your arm forward, pulling the band. Slowly return.

Repeat 3 x 15-25 times.

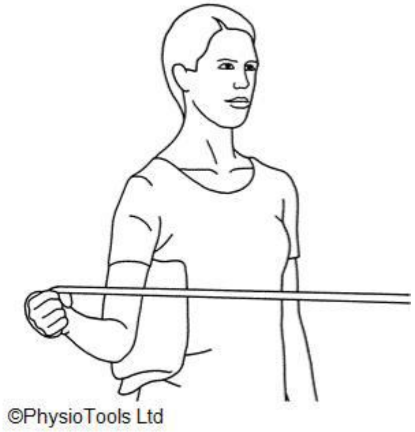

Stand or sit. Place a rolled towel between your upper arm and side. Your upper arm should be slightly forward and your elbow at a right angle. Hold an exercise band. Pull the band taking your forearm out 45 degrees. Hold for 5 secs.

Repeat 3 x 15-25 times.

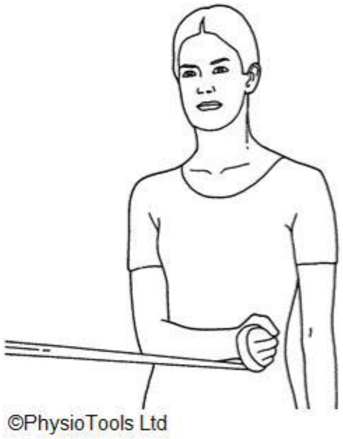

Stand keeping your upper arm close to the side and elbow at a right angle. Hold a rubber exercise band. Pull the band toward your stomach and hold for 5 secs.

Repeat 3 x 15-25 times.

BMJ Publishing Group Limited (BMJ) disclaims all liability and responsibility arising from any reliance placed on this supplemental material which has been supplied by the author(s)

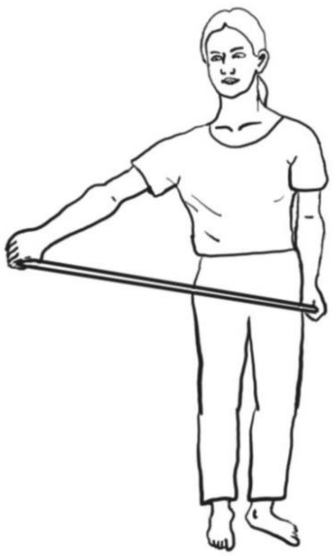

Hold an exercise band with both hands. Pull the band outwards to about 45-60 degrees with back of your hand leading the movement. Hold for 5 secs.

Repeat 3 x 15-25 times

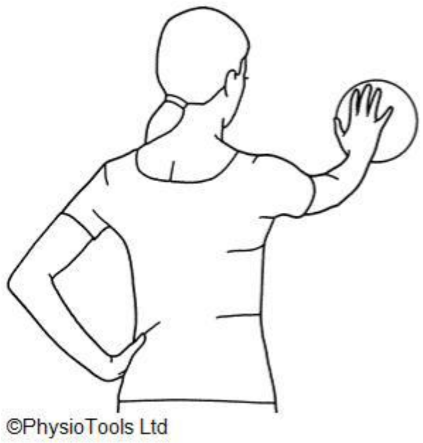

Stand with one foot forward. Keep your back straight. Place the palm of your hand against a wall using a ball or a piece of cloth. Lean your body weight onto your hand. Control your shoulder position and move hand in small horisontal motions.

Repeat 3 x 15-25 times.

32

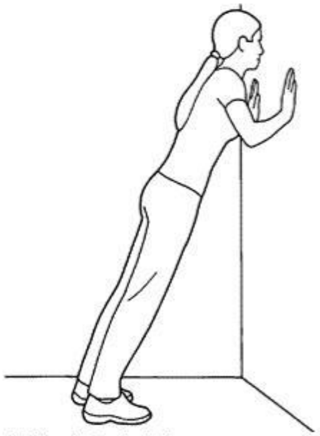

Stand facing a wall with your arms straight and hands on the wall. Do push-ups against the wall keeping your body in a straight line.

Repeat 3 x 15-25 times.

Phase IV (9-12 weeks)

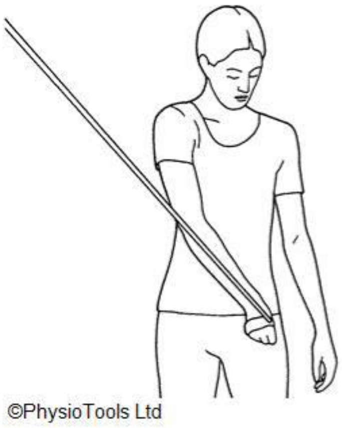

Stand with your arm up and out to the side. Hold a rubber exercise band. Pull the band down and across your body letting your thumb lead the movement.

Repeat 3 x 15-25 times.

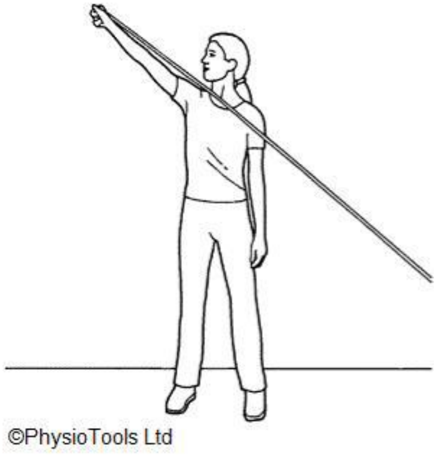

Stand or sit with the hand of the arm to be exercised on your opposite hip. Hold on to a rubber exercise band. Pull the band up towards the opposite side.

Repeat 3 x 15-25 times.

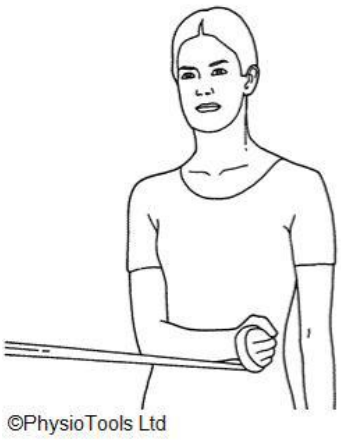

Stand keeping your upper arm close to the side and elbow at a right angle. Hold a rubber exercise band. Pull the band toward your stomach.

Repeat 3 x 15-25 times.

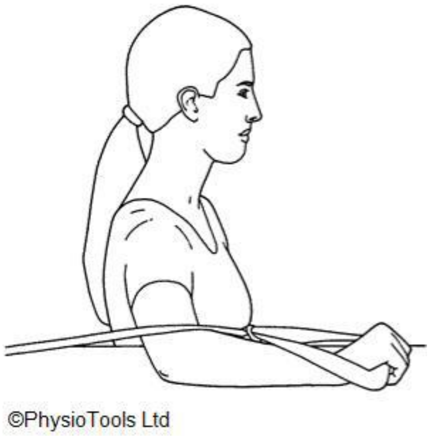

Alternatively,
Sit on a chair with your arm lifted to the side and your elbow at a right angle supported on a table. Hold on to a rubber exercise band which is fastened behind you. Pull the band keeping elbow bent and resting on the table.

Repeat 3 x 15-25 times.

BMJ Publishing Group Limited (BMJ) disclaims all liability and responsibility arising from any reliance placed on this supplemental material which has been supplied by the author(s)

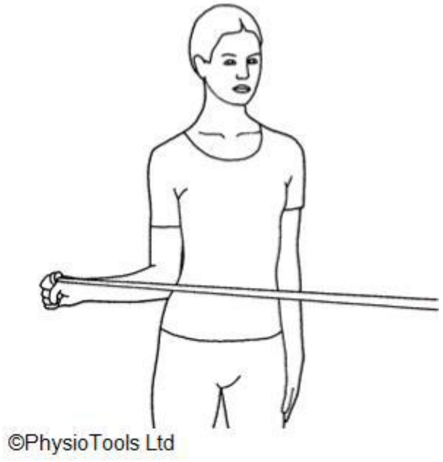

Stand keeping your upper arm close to the side and elbow at a right angle. Hold a rubber exercise band. Pull the band by turning your forearm outwards.

Repeat 3 x 15-25 times.

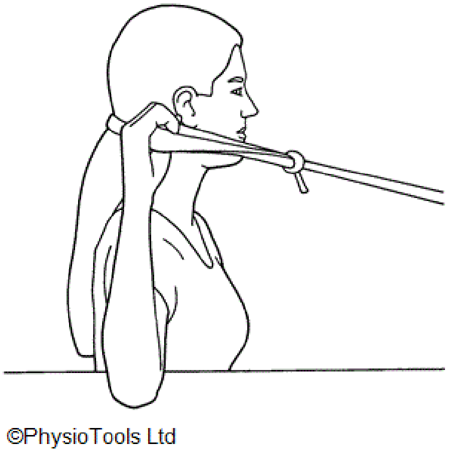

Alternatively,
Sit on a chair with your arm lifted out to the side and elbow at a right angle supported on a table. Hold on to a rubber exercise band which is in front on you. Pull the band keeping your elbow bent and resting on the table.

Repeat 3 x 15-25 times.

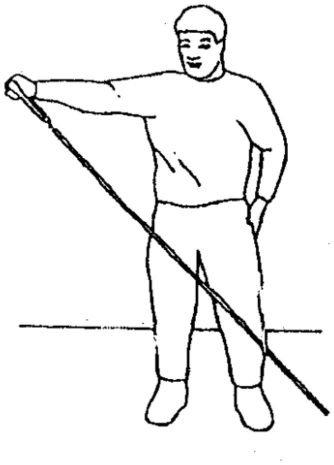

© PhysioTools Ltd

Stand or sit with the hand of the arm to be exercised at your side. Hold on to a rubber exercise band attached at floor level. Pull the band up upwards up to 90 degrees.

Repeat 3 x 15-25 times.

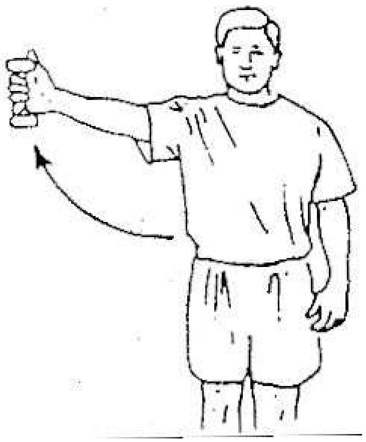

© PhysioTools Ltd

Stand or sit with the hand of the arm to be exercised at your side. Lift hand in thump up position up to a 90° of abduction.

Repeat 3 x 15-25 times.

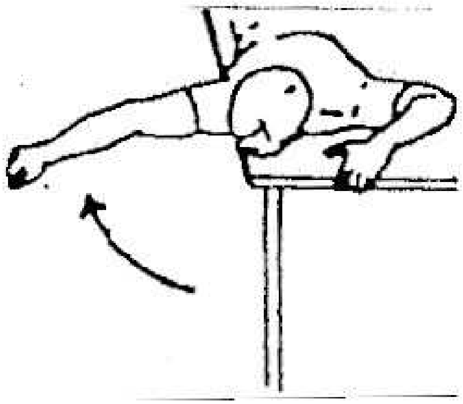

© PhysioTools Ltd

Lying face down with your arms out to the side. Lift your arm toward the ceiling with back of your hand leading the movement.

Repeat 3 x 15-25 times.

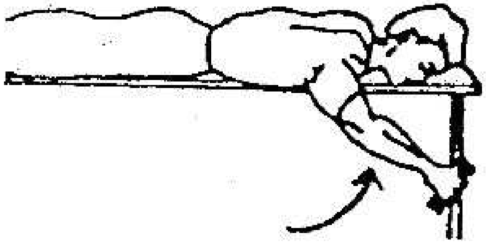

© PhysioTools Ltd

Lying face down with your arms out to the side. Lift your arm forwards up to 100° of flexion with your thumbs leading the movement.

Repeat 3 x 15-25 times.

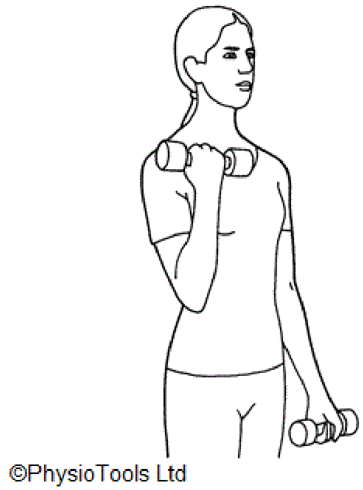

Stand with arms hanging down. Hold a hand weights and turn your palms forward. Bend alternate elbows briskly.

Repeat 3 x 15-25 times.

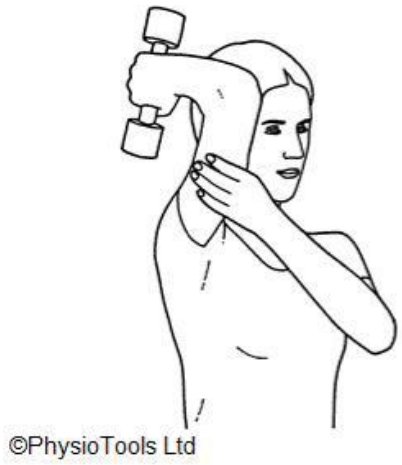

Sit or stand holding a kg hand weight. Bring the arm to be exercised up with the elbow pointing to the ceiling. Support the elbow with the other hand. Straighten the arm holding the weight.

Repeat 3 x 15-25 times.
